# Effects of ginger-loaded chitosan nanoparticles on growth, morphological and biochemical attributes of *Sesamum indicum* L.

**DOI:** 10.1371/journal.pone.0349701

**Published:** 2026-07-02

**Authors:** Hunaira Nasreen, Abdul Ghaffar, Sumaira Yousaf

**Affiliations:** 1 Department of Biochemistry, Government College University, Faisalabad, Pakistan; 2 Nuclear Institute for Agriculture & Biology, Faisalabad, Pakistan; National Cancer Institute at Frederick, UNITED STATES OF AMERICA

## Abstract

Chitosan nanoparticles (ChNPs) have gained attention due to their biodegradability, biocompatibility, and non-toxicity, and are found in a wide range of agricultural products. The green synthesis and characterization of ChNPs using ginger extract were evaluated for their effects on biochemical and morphological parameters in sesame (*Sesamum indicum* L.). Ginger-loaded ChNPs synthesis, size, structure, morphology, and crystallinity were confirmed via UV-Vis spectrophotometry, DLS (151.7 nm, ± 30 mV), FTIR, SEM, and XRD, respectively. Foliar applications of ChNPs (50 mg L^-1^ and 100 mg L^-1^) and pesticides confidor (2.5 mL L^-1^) confidor + talstar (2.5 mL L^-1^) were applied at flowering and capsule stages under controlled conditions. ChNPs at 100 mg L ⁻ ¹ significantly enhanced plant height 5.3%, leaf area 26.5%, number of capsules 43.6%, capsule weight (12.4%), stem diameter 34.4%, total chlorophyll 11.9%, and catalase activity 53.1% compared with the control (P < 0.05), while reducing peroxidase 85.7% and PAL 59%. In contrast, confidor and confidor + talstar treatments showed compromised effects. Present research demonstrated that green synthesized ChNPs have good potential for use in agriculture and can significantly increase sesame growth and yield.

## Introduction

Growing global food security challenges demand sustainable crop improvement strategies to meet rising demands for nutritious oils and proteins. *S. indicum* L. is one of the oldest oilseed crops but suffers from low yield and susceptibility due to biotic and abiotic stresses [1]. Chemical fertilizers and pesticides have increased yields in the short term but degrade soil, harm human health, and cause environmental pollution, requiring green technology for better growth and resilience [2,3].

Chitosan nanoparticles (ChNPs) gained interest as biodegradable, biocompatible carriers, plant growth boosters, defense inducers, and smart nano-delivery systems for agrochemicals [4,5]. ChNPs enhance seed germination, seedling vigor, photosynthetic capacity, nutrient uptake, and yield in a variety of crops, all while activating antioxidant and defense enzymes and lowering stress damage [6–8]. ChNPs perform better than bulk chitosan, owing to their larger surface area, better adsorption to seed surfaces, improved leaf absorption, and ability to release encapsulated bioactive chemicals under controlled conditions.

Ginger (*Zingiber officinale*) extracts high in phenolic compounds and terpenoids have strong antioxidant, antibacterial, and anti-inflammatory properties and are widely used in pharmaceutical and food systems [9,10]. However, their low aqueous stability and high volatility under environmental conditions severely restrict direct agricultural application. Encapsulating ginger extracts or oils in ChNPs improves their stability, regulates release, and increases antibacterial and antioxidant efficacy in food preservation, medicine, and nanobactericide applications [11,12].

Ginger-loaded ChNPs are a promising two-in-one agronanotechnology. Chitosan promotes growth and induces defense, while ginger provides antioxidant and antibacterial properties [13,14]. ChNPs could (i) promote early growth and photosynthetic capacity, (ii) activate endogenous antioxidant and osmoprotective pathways, and (iii) restrict pathogen pressure, all of which improve total plant performance under both ideal and stress conditions. Despite the rapid proliferation of ChNP-based nanofertilizers and nanopesticides, present agricultural applications primarily focus on unloaded chitosan, mineral-loaded ChNPs, or vitamin/fulvic acid carriers in a variety of crops [14,15].

A major development in agronanotechnology has been the functionalization of ChNPs with bioactive carriers, which converts them into smart nanocarriers for vitamins, micronutrients, and traditional treatments. ChNPs loaded with thiamine in chickpea enhance germination, growth, and development. Production of auxin (IAA) and activation of defense enzymes and reduction of cell death caused by pathogens, transforming them into growth stimulators and defense activators [16]. Chitosan-selenium nanoparticles in bitter melon and citrus, as well as fulvic acid-releasing ChNPs in soybean, improve growth and yield while also altering ion homeostasis, antioxidant machinery, and stress-marker gene expression, resulting in increased salt tolerance [17,18].

ChNPs coatings and sprays can enhance germination, root architecture, pigments, and osmolytes. However, no study has explored how phytochemical-loaded ChNPs affect the overall growth, morphology, and biochemistry of sesame [8,19,20]. This work aims to investigate the impact of ginger-loaded chitosan nanoparticles on growth, morphology, and biochemistry in *S. indicum* L.

## Material and methods

### Preparation of ChNPs encapsulating ginger extract

The fresh ginger was obtained from a local vegetable shop in Faisalabad, Pakistan. Ginger rhizome was washed, dried, and then crushed into fine powder. Ginger powder 5 g/100 mL was stirred for 2 hours at 40 ºC in 100 mL of distilled water, filtered, and kept at 4 ºC for further use. Chitosan (1% w/v) was dissolved in 1% acetic acid by magnetic stirring for 2 hours. The pH was adjusted to 4.8 using 10% sodium hydroxide. Ginger extract (100 mL) was added to the aforesaid solution, and it was stirred for 2 hours. The sodium tripolyphosphate (STPP) solution (0.2% w/v) was then added dropwise with continuous stirring for 2 hours. The solution was centrifuged for 20 minutes at 15,000 rpm. The resulting pellets were washed with water and lyophilized to obtain ChNPs encapsulating ginger extract.

### Characterization of chitosan nanoparticles

The hydrodynamic diameter and polydispersity index (PDI) of the ChNPs were determined using a Zetasizer Nano ZS90 (Malvern Instruments, Malvern, UK) with dynamic light scattering, and the zeta potential value (mV) was calculated. At a constant temperature of 25 ºC and a fixed angle of 90°, the detector was used to monitor the nanoparticle solutions at least three times.

Biologically reduced chitosan nanoparticles were analyzed using a UV-vis spectrophotometer to confirm the synthesis of ChNPs (Evolution™260 Bio UV–visible, Thermo Fisher Scientific) in the 200–800 nm range.

FTIR analyses were performed using the VERTEX 70v from Bruker Optics to determine the functional groups of ChNPs. A multi-zone nitrogen purge-equipped JASCO FTIR 4100, Yorkshire WF160 PR, UK, was used for Fourier transform infrared spectroscopy examination with a resolution of 4 cm^−1^; spectra were recorded between 4000 and 400 cm^-1^ [21].

ChNPs were morphologically evaluated using a scanning electron microscope (Zeiss Sigma 300, Zeiss, Germany) operated at 3 kV. The nanoparticles were ultrasonicated for 10 minutes and were dried on silicon slides at room temperature. The samples were sputtered on a gold film to get clear images. The values of diameter were then imported into Origin software (OriginPro Learning Edition, OriginPro 2025b) for the statistical analysis of average size and the plotting of the size distribution histogram [22].

XRD pattern of ChNPs analyzed using an X-ray diffractometer (Bruker D2 Phaser 2nd Gen). Cu Kα radiation (wavelength of 0.1541 nm) was used to record the spectra, which ranged from 2θ = 10-80 ° diffraction angles [23].

### Foliar application of ChNPs and pesticides in a completly randomized design on sesame

The soil was collected from one of the experimental fields of the Nuclear Institute for Agricultural & Biology (NIAB). The collected soil was equally divided (20 kg per pot) into 75 experimental pots (45 cm x 30 cm) for planting. The soil texture is silt loam (4.65% sand, 68.34% silt, 27.01% clay) with pH 6.2, temperature 27 ⁰C, and relative humidity 68% at the time of sowing. Five genetically different varieties of sesame, resistant NIAB Pearl (N-Pearl), NIAB Millennium (N-Millennium), NIAB Sesame 2016 (NS-2016), susceptible (TS-5, TH- 6) were planted in a completely randomized design (CRD) in pots at NIAB (31.4167° N, 73.0833° E) in Faisalabad, Punjab, Pakistan, under controlled conditions. The pots were watered at 45, 70, and 90 days after sowing. The experiment consisted of five treatments: two concentrations of ChNPs (50 and 100 mg L ⁻ ¹), the pesticides confidor (2.5 mL L ^−1^) confidor+talstar (2.5 mL L^-1^), and an untreated control. All these treatments were applied in triplicate in the CRD pot experiment. ChNPs two concentrations (50 and 100 mg L ⁻ ¹) foliar applications were made throughout the two main growth phases such as flowering (51 days after sowing) and capsule formation (82 days after sowing). Similarly confidor (46 and 80 days after sowing) confidor+talstar (46 and 80 days after sowing) and by using a calibrated knapsack sprayer [24]. The effectiveness of confidor (Imidacloprid 20% SL) against common sesame pests (whitefly) was assessed. A treatment of confidor+talstar (bifenthrin) was added to evaluate synergistic effects.

### Morphological and biochemical analysis of sesame leaves

Morphological data were collected in the pot experiment to evaluate plant growth. Plant height, number of leaves, and root length were measured by a leaf area meter and digital callipers. Leaf samples were taken from each treatment group for biochemical examination and kept at −20 °C to preserve metabolite stability [25].

Biochemical parameters such as catalase (CAT), polyphenol oxidase (PPO), phenylalanine ammonia-lyase (PAL), peroxidase (POD), and chlorophyll (Chl a, Chl b, and total Chl) were evaluated in sesame leaves. Catalase activity was determined by grinding Sesame leaves 0.5 g into a fine paste with sodium phosphate buffer, 50 mL, with 7.0 pH in a pre-refrigerated pestle and mortar. The homogenate was centrifuged for 20 minutes at 14,000 rpm, and the supernatant was collected and kept at 4 °C. Phosphate buffer 50 µL, substrate 50 µL, and 2 µL of ground leaf sample made up the reaction mixture in the eppendorf. Absorbance values were taken at 240 nm wavelength for 0 and 1 minute by using a UV-vis spectrophotometer (Evolution™260 Bio UV-visible, Thermo Fisher Scientific) [26].

Phenylalanine ammonia lyase (PAL) was extracted from 0.5 g fresh leaves ground in 0.2 M sodium borate buffer (pH 8.7) at 4 ºC, then centrifuged at 12,000 x g for 15 minutes. For the assay, 125 µ moles L-phenylalanine, sodium borate buffer, and enzyme extract (total 1500 µL) were incubated at 40 ºC for 60 minutes, followed by 50 µL 5 *N* HCl. Absorbance was measured at 290 nm using a UV-visible spectrophotometer [27].

Peroxidase (POD) was extracted from sesame fresh leaves (0.55 g) grinding with a phosphate buffer 280 µL, pH 7.0 was used. The activity of POD was assessed using the supernatant after the extract was centrifuged at 10,000 rpm for 20 minutes at 4 ºC. The addition of diluted enzyme extract to phosphate buffer started the process. After one minute of monitoring oxidation at 470 nm, the enzyme activity was computed. This value was deducted from those containing enzyme extract using the reaction mixture without enzyme extract as a blank. The unit of POD activity was µmol min^-1^ g^-1^ [28].

Fresh leaves were ground with phosphate buffer to create the enzyme extract, which was then centrifuged at 10,000 x g for 15 minutes at 4 ºC. The soluble polyphenol oxidase activity was measured in the supernatant. Reaction mixture that included 50 µL of phosphate buffer (pH 7.0), 50 µL of substrate, and enzyme extract in a cuvette. As soon as the enzyme was added, absorbance variations at 280 nm were recorded at 0 and 1 minute.

Chlorophyll a and chlorophyll b in the leaves were estimated by using standard protocols [29]. Fresh leaves (0.5 g) from each treatment were macerated in 80% acetone, and 2 mL solution was kept at −4 °C for 24 hours. [30]. Absorbances were measured at 645 and 663 nm.


$$\begin{array}{c} \text{Chlorophyll a }(\text{mg}/\text{g})=\;(\text{12}.\text{7 }\times \text{ A663})-\;(\text{2}.\text{59 }\times \text{ A645}) \end{array}$$ (1)

$$\begin{array}{c} \text{Chlorophyll b }(\text{mg}/\text{g})=\;(\text{22}.\text{9 }\times \text{ A645})-\;(\text{4}.\text{7 }\times \text{A663}) \end{array}$$ (2)

$$\begin{array}{c} \text{Chlorophyll total }(\text{mg}/\text{g})=\;(8.2\;\times \;A663)+\;(20.2\;\times A645) \end{array}$$ (3)

### Statistical analysis

Two-way ANOVA as a statistical model was used to determine the effect and variance of different concentrations of nanoparticles and pesticides on sesame plants. The p-value was taken to be significant when it was ≤ 0.05. RStudio 4.5.2 was used to make spider graphs, which were communicated by comparing [(Treatment−control)/control]×100. Principal component analyses (PCA) were assessed to determine the correlation of different morphological and biochemical parameters using SRPLOT.

## Results and discussion

### Biosynthesis of ginger-loaded chitosan nanoparticles

The chitosan solution and the extract from ginger rhizome (*Zingiber officinale)* formed a stable, light-yellow colloidal mixture, indicating the formation of complexes. UV–visible spectrophotometer used to analyze the nanoparticle, a single sharp peak at 296 nm (Fig 2a) indicated the synthesis of chitosan ginger-loaded nanoparticles. The ginger extract facilitates the formation of nanoparticles by acting as a precursor and reducing agent [31]. Ginger extract molecules bind inside the resulting nanoparticle matrix when the anionic phosphate group of STPP binds to the cationic NH_2_ groups of chitosan and transforms them into chitosan nanoparticles [32]. This aligns with previous research representing that plant extracts can act as both reducing and stabilizing agents in nanoparticle synthesis, enhancing their biocompatibility and functionality [33]. The biosynthesis of ginger-loaded chitosan nanoparticles is a significant advancement in developing eco-friendly, biologically active nanomaterials for agricultural applications.

**Fig 1:**
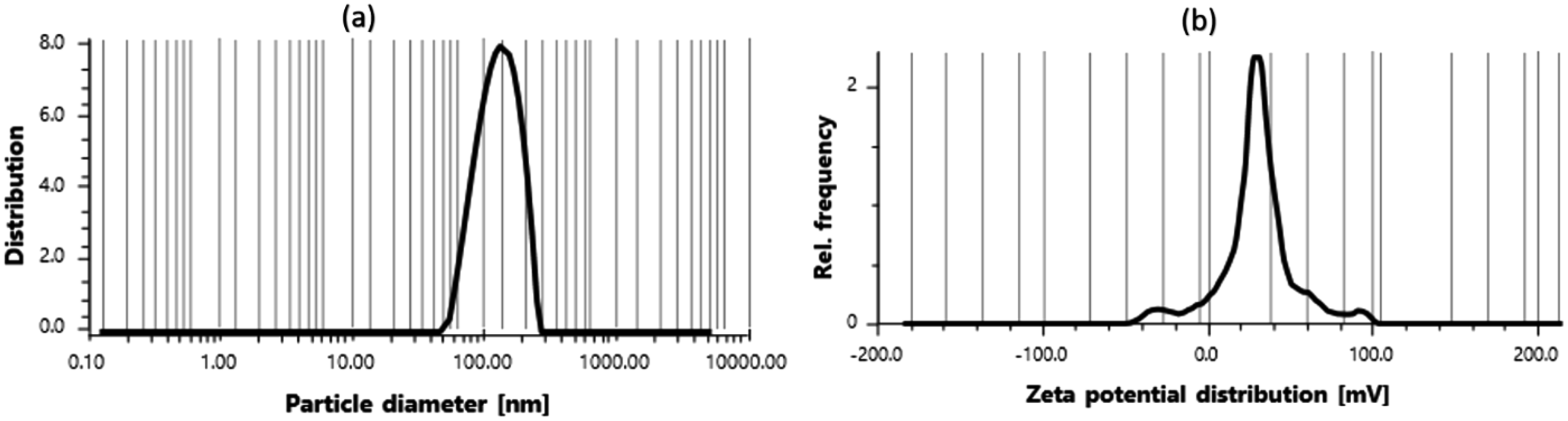
Characterization of ginger-loaded chitosan nanoparticles, (a) Zeta sizer analysis and (b) zeta potential analysis.

### Dynamic light scattering analysis of ginger-loaded chitosan nanoparticles

The zeta sizer showed the nanoparticle’s diameter was 151.7 nm, and its Polydispersity Index (PDI) value was 0.42 (Fig 1a). A PDI value in the range (0.3–0.5) indicates a relatively uniform particle size distribution with moderate polydispersity. Chitosan nanoparticles formation occurs through ionic gelation by electrostatic interactions between the positively charged amino groups of chitosan and negatively charged cross-linking agents such as STPP. A zeta potential of at least ±30 mV is generally required for physically stable nanoparticle suspensions, as it creates enough electrostatic repulsion to prevent aggregation. Because of the repulsion, a large negative or positive zeta potential prevents nanoparticles from aggregating and provides stability. The biosynthesized ChNPs were found to have a zeta potential of +31.4 mV (Fig 1b), which exceeds this threshold and indicates a strong positive surface charge. Significant electrostatic repulsion between nanoparticles may be demonstrated by higher positive zeta potential, which inhibits aggregation in the aqueous phase. These results indicate that monodispersed stable ChNPs are produced by utilizing ginger extract [10].

### Fourier transform infrared spectrophotometry analysis of ginger-loaded chitosan nanoparticles

**Fig 2:**
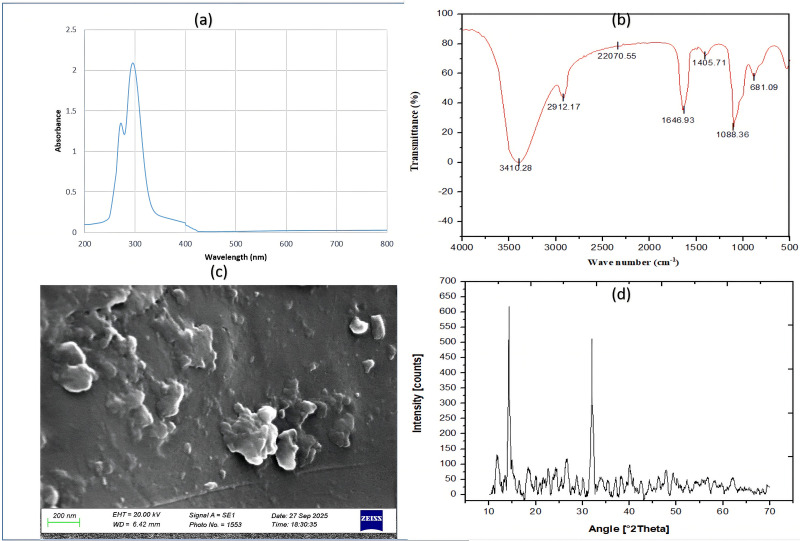
Physicochemical and morphological characterization of ginger-loaded chitosan nanoparticles, (a) UV–vis spectrophotometer analysis, (b) FTIR analysis, (c) SEM analysis, and (d) XRD analysis.

**Fig 3:**
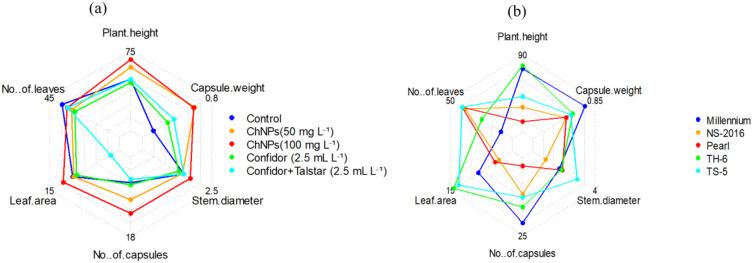
Spider plot of all the(a) treatments and (b) varieties on the morphological attributes of sesame plants.

FTIR spectra of ChNPs in the 4000−500 cm^−1^ region, providing insight into their functional groups (Fig 3b). A peak at 3410 cm^−1^ was detected due to the overlap of OH and NH stretching. The C-H stretch peak at 2927 cm^−1^ shows the chitosan structure. The peak at 1644 cm^-1^ indicated the presence of a C = O bond of the amide group. The 1405 cm peak reflects chitosan binding to gingerol, a glycosidic bond was detected at 1088 cm^-1^ (C-O stretch), and the peak at 681 indicates a phosphate group (Fig 2b). Glycosidic and amide linkages, along with the peaks associated with gingerol, indicate stable encapsulation of gingerol, which is responsible for the bioactivity of ChNPs [34].

### Scanning electron microscope analysis of ginger-loaded chitosan nanoparticles

Surface morphology of chitosan nanoparticles loaded with ginger extract, imaged at 20 kV using a 6.4 mm working distance and 200 nm scale bar (Fig 2c). The image shows clustered nanoscale structures with irregular rough surface topology, resembling the natural self-assembly of chitosan polymers that contain phytochemicals [35]. Clear quasi-spherical and amorphous groups appear, pointing to irregular nucleation and growth in nanoparticle development. Measurements indicate sizes from roughly 50–150 nm (average ~100 nm, n = 50 particles analyzed in ImageJ from the image), where most forms stay under the 200 nm scale bar as grouped clusters or isolated nanostructures against the matrix. These features, statistically shown as a narrow size distribution (SD ± 30 nm), are characteristic of ChNPs and favorable for bioactive encapsulation and release, as the rough, porous surface enhances biological interactions [21].

### X-ray diffraction analysis of ginger-loaded chitosan nanoparticles

The XRD pattern (Fig 2d) of chitosan nanoparticles shows that it has large peaks at 10° 2θ (020 reflection) and 20° 2θ (110/200 reflection) with a small peak at 32°, which is a semi-crystalline structure with mostly amorphous regions. Nanoscale crystallite sizes of 8 nm (range 510 nm) are obtained by peak broadening (FWHM 0.7–1.5° or β 0.0175 rad) using the Scherrer equation D = Kλ / (β cos θ) (D=0.94×0.15406βcosθ) D=βcosθ0.94×0.15406), which is a sign of disorder due to the synthesis of ionic gelation. This is a lower crystallinity than the bulk chitosan pattern that is compatible with ionic gelation and is appropriate in agricultural applications that require flexibility and biodegradability [36].

### Effect of biosynthesized ginger-loaded ChNPs on the morphological attributes of sesame

**Fig 4:**
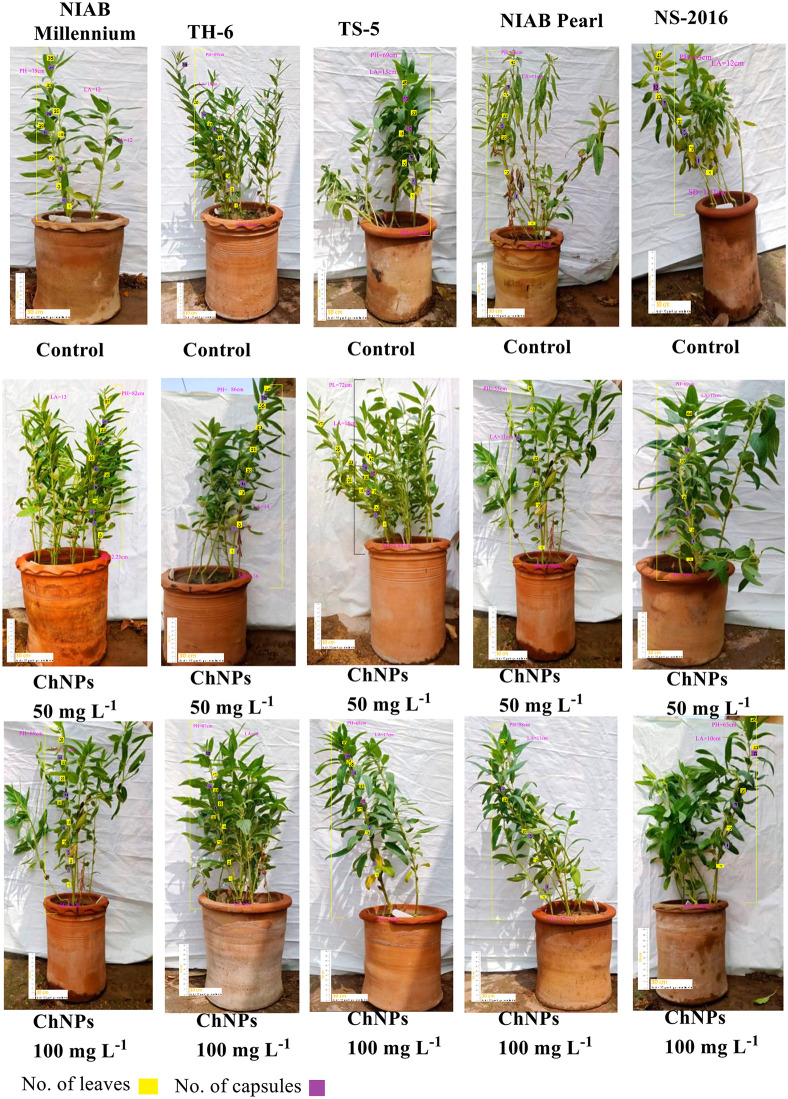
Influence of biosynthesized chitosan nanoparticles on morphological attributes of sesame.

Control (untreated) plants are least effective with the lowest plant height, capsule number, leaf area, and capsule weight (Fig 4). Both concentrations of ChNPs significantly improved vegetative and reproductive characteristics, producing more balanced profiles in spider plots (Fig 3a and b). ChNPs at 50 mg L^-1^ enhanced plant height by 3.3%, number of leaves by 21.5%, leaf area by 9.8%, and capsule number by 25.0%, compared to the untreated control. The weight of the capsule decreased 7%, and the stem diameter 5%. At 100 mg L^-1^, the concentration-dependent improvements were greater; plant height was improved by 5.3%, leaf area by 26.5%, the number of capsules by 43.6%, the weight of capsules by 12.4%, and the stem diameter by 34.4%. These improvements in morphological parameters were statistically significant (Table 1) (P < 0.05). These outcomes align with tomato, where foliar ChNPs up to 3000 mg L^-1^ enhanced plant height and stem diameter and yield and fruit quality compared to untreated plants [37], and with maize, where 0.01–0.12% Cu chitosan NPs enhanced plant height, stem diameter and chlorophyll content, with obvious superiority over bulk chitosan, CuSO_4_ and fungicide treatments [38]. The promotion of growth by ChNPs has been extensively explained by their nanoscale characteristics, which increase adhesion, controlled release and uptake of bioactive chitosan, resulting in increased photosynthetic pigments (usually 20−60% increase), enhanced nutrient uptake, and enhanced expression of auxin related genes and endogenous IAA biosynthesis, as demonstrated in wheat seedlings treated with 5 µg mL ^−1^ ChNPs [39]. The enhanced activity of ChNPs is due to their nanoscale properties, which regulate the release of bioactive chitosan and stimulation of endogenous growth pathways, including increased photosynthesis and nutrient uptake, without causing stress responses. On the other hand, the chemical pesticide treatments produced fewer morphological profiles, which is indicative of low biostimulatory activity. Confidor (2.5 mL L^-1^) had a negative effect on plant height 0.9%, capsule weight 9.4%, and a positive effect on leaf number 12.0% and capsule number 5.7%, leaf area and stem diameter were similar to the control (statistically non-significant, P > 0.05). Morphological traits have been reported to be depressed or phytotoxic by conventional pesticides: mancozeb and chlorpyrifos decreased root and shoot length, seedling vigor, and tolerance index in *Allium cepa*, and the degree of oxidative stress and reduced growth were closely linked with increasing pesticide concentration Confidor sprays increased plant height and leaf number in garlic, although some treatments decreased fresh biomass relative to control, suggesting that effects are crop and dose-dependent and not always growth promoting. The confidor + talstar mixture had stronger negative impacts on various parameters: plant height was reduced by 2.5%, leaf area by 19.4%, capsule number by 12.7%, stem diameter by 16.5%, and capsule weight by 13.5%, although there was an increase of 1% in 1% no. of leaves. ChNPs produced uniform improvements in morphological characteristics, but chemical pesticides produced compromised effects. The morphological profiles were different in varietal responses to all treatments. N- Millennium had the highest plant height 18.7% number of capsules 65% and stem diameter of stem 5.5% than the control, and moderate leaf area 2.7% but lower number of leaves, 8.0%. N- Pearl had the least plant height 21%, number of capsules 47% and leaf area 8%. TH-6 had the greatest plant height 21%, leaf area 19%, and capsule number 33.5, and equal weight of capsules. TS-5 had the greatest stem diameter 45.0%, number of leaves 26% and leaf area 16% but intermediate other characteristics. This shows that the potential biostimulant application of chitosan nanoparticles in enhancing balanced morphological growth of sesame plants, and possibly eliminating the need to use chemical pesticides to sustain agriculture.

**Table 1:** Effect of ginger-loaded ChNPs on morphological attributes (Mean ± SE, n = 15).

Treatment	N-Millennium	NS-2016	N-Pearl	TH-6	TS-5
**Plant height (cm) treatment x variety interaction Mean±SE** (LSD = 1.662)					
**Control**	79.33 ± 0.33^f^	63.33 ± 0.88^g^	56.00 ± 1.00^b^	81.33 ± 0.88^l^	68.33 ± 0.33^m^
**ChNPs(50 mg L ⁻ ¹)**	85.00 ± 0.58^j^	67.67 ± 0.67^m^	53.33 ± 0.33^c^	85.67 ± 0.33^i^	71.33 ± 0.67^e^
**ChNPs(100 mg L ⁻ ¹)**	90.00 ± 0.58^a^	63.67 ± 0.67^g^	58.00 ± 0.00^h^	88.00 ± 0.58^j^	79.33 ± 0.67^f^
**Confidor (2.5 mL L ⁻ ¹)**	69.33 ± 0.33^g^	56.67 ± 0.33^c^	50.00 ± 0.58^d^	87.00 ± 1.00^i^	67.33 ± 0.88^m^
**Confidor+talstar (2.5 mL L ⁻ ¹)**	81.00 ± 0.00^l^	60.33 ± 0.33^k^	59.33 ± 0.33^h^	80.33 ± 0.33^f^	58.67 ± 0.33^c^
**No. of leaves treatment x variety interaction Mean±SE** (LSD = 1.766)					
**Control**	35.00 ± 0.58^b^	37.00 ± 0.58^a^	34.67 ± 0.88^e^	39.67 ± 0.33^d^	36.67 ± 0.67^a^
**ChNPs(50 mg L ⁻ ¹)**	44.33 ± 0.88^g^	43.33 ± 0.67^e^	46.67 ± 0.33^h^	41.00 ± 0.58^d^	47.00 ± 1.00^f^
**ChNPs(100 mg L ⁻ ¹)**	41.67 ± 0.67^d^	45.00 ± 0.00^g^	44.33 ± 0.88^g^	42.33 ± 0.33^e^	46.00 ± 1.15^h^
**Confidor (2.5 mL L ⁻ ¹)**	32.33 ± 0.33^c^	47.00 ± 0.58^f^	42.33 ± 0.33^d^	37.33 ± 0.33^a^	46.00 ± 0.58^g^
**Confidor+talstar (2.5 mL L ⁻ ¹)**	39.00 ± 0.00^a^	42.33 ± 0.67^d^	47.00 ± 0.58^f^	33.33 ± 0.33^c^	41.33 ± 0.67^d^
**Leaf area (cm) treatment x variety interaction Mean±SE** (LSD = 0.781)					
**Control**	12.00 ± 0.00^h^	11.01 ± 0.33^g^	8.33 ± 0.33^a^	15.67 ± 0.33^e^	15.00 ± 0.00^c^
**ChNPs(50 mg L ⁻ ¹)**	13.00 ± 0.58^f^	11.63 ± 0.33^h^	11.07 ± 0.33^g^	16.67 ± 0.33^b^	15.67 ± 0.33^e^
**ChNPs(100 mg L ⁻ ¹)**	14.67 ± 0.33^c^	13.33 ± 0.33^f^	15.40 ± 0.00^e^	18.00 ± 0.00^d^	17.00 ± 0.00^b^
**Confidor (2.5 mL L ⁻ ¹)**	11.00 ± 0.00^g^	11.33 ± 0.33^i^	11.03 ± 0.00^h^	13.43 ± 0.33^f^	13.67 ± 0.33^c^
**Confidor+talstar (2.5 mL L ⁻ ¹)**	11.00 ± 0.00^i^	10.67 ± 0.33^g^	11.01 ± 0.00^i^	6.00 ± 0.00^a^	11.33 ± 0.33^h^
**No. of capsules treatment x variety interaction Mean±SE** (LSD = 1.694)					
**Control**	16.00 ± 1.00^e^	12.67 ± 0.67^c^	7.90 ± 0.58^i^	15.00 ± 1.00^f^	14.33 ± 0.88^c^
**ChNPs(50 mg L ⁻ ¹)**	23.33 ± 0.33^a^	15.00 ± 0.58^f^	13.33 ± 0.33^c^	17.67 ± 0.88^g^	13.00 ± 0.58^b^
**ChNPs(100 mg L ⁻ ¹)**	21.33 ± 0.67^b^	17.00 ± 0.58^e^	17.00 ± 0.00^g^	24.33 ± 0.33^a^	15.00 ± 0.00^f^
**Confidor (2.5 mL L ⁻ ¹)**	18.33 ± 0.33^g^	12.67 ± 0.33^c^	14.67 ± 0.67^f^	13.00 ± 0.58^c^	11.00 ± 0.58^i^
**Confidor+talstar (2.5 mL L ⁻ ¹)**	12.57 ± 0.33^c^	15.93 ± 0.88^e^	6.33 ± 0.33^d^	10.00 ± 0.58^i^	12.67 ± 0.33^c^
**Diameter of stem (cm) treatment x variety interaction Mean±SE** (LSD = 0.095)					
**Control**	2.30 ± 0.02^h^	1.48 ± 0.02^c^	2.51 ± 0.02^g^	2.38 ± 0.05^i^	2.23 ± 0.01^h^
**ChNPs(50 mg L ⁻ ¹)**	2.57 ± 0.02^b^	1.81 ± 0.01^a^	1.41 ± 0.01^c^	1.49 ± 0.04^c^	3.00 ± 0.02^b^
**ChNPs(100 mg L ⁻ ¹)**	3.33 ± 0.04^f^	3.24 ± 0.02^e^	2.45 ± 0.03^g^	2.36 ± 0.04^b^	3.27 ± 0.06^f^
**Confidor (2.5 mL L ⁻ ¹)**	1.30 ± 0.02^c^	1.53 ± 0.03^c^	2.32 ± 0.05^i^	2.43 ± 0.06^g^	3.11 ± 0.03^e^
**Confidor+talstar (2.5 mL L ⁻ ¹)**	1.30 ± 0.04^a^	1.63 ± 0.02^d^	1.41 ± 0.01^c^	2.37 ± 0.04^i^	2.38 ± 0.03^h^
**Weight of capsule(g) treatment x variety interaction Mean±SE** (LSD = 0.082)					
**Control**	0.82 ± 0.00^a^	0.74 ± 0.05^b^	0.86 ± 0.02^c^	1.02 ± 0.0^d^	0.92 ± 0.03^e^
**ChNPs(50 mg L ⁻ ¹)**	0.81 ± 0.03^c^	0.87 ± 0.02^a^	0.90 ± 0.01^e^	0.74 ± 0.03^c^	0.73 ± 0.02^b^
**ChNPs(100 mg L ⁻ ¹)**	1.50 ± 0.03^f^	0.70 ± 0.02^b^	0.81 ± 0.02^a^	0.80 ± 0.02^c^	1.09 ± 0.04^d^
**Confidor (2.5 mL L ⁻ ¹)**	0.74 ± 0.04^b^	0.76 ± 0.02^c^	0.92 ± 0.01^e^	0.81 ± 0.01^a^	0.72 ± 0.04^b^
**Confidor+talstar (2.5 mL L ⁻ ¹)**	0.76 ± 0.02^c^	0.73 ± 0.04^e^	0.79 ± 0.05^c^	0.73 ± 0.02^b^	0.76 ± 0.03^a^

Means sharing similar letters in a row or in a column are statistically non-significant (P > 0.05).

### Effect of biosynthesized ginger-loaded ChNPs on biochemical attributes of sesame

**Fig 5:**
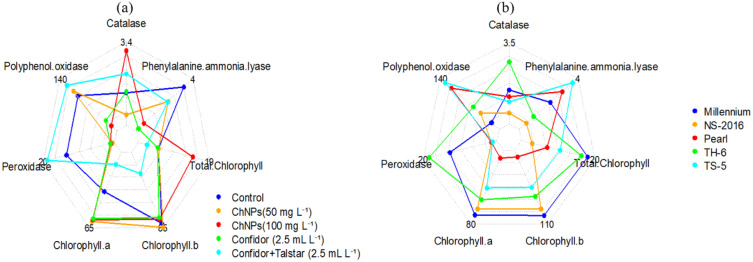
Spider plot of all the (a) treatments and (b) varieties on the biochemical attributes of sesame plants.

**Fig 6:**
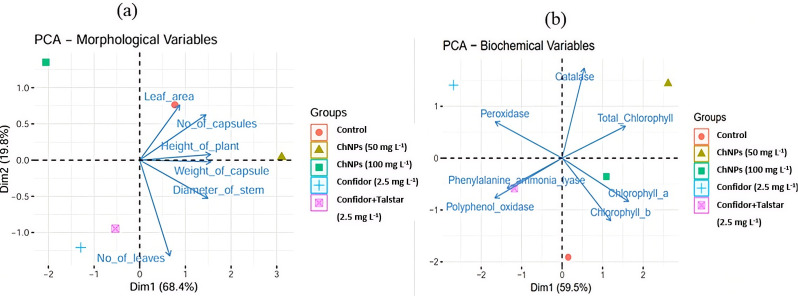
Correlation between (a) morphological and (b) biochemical attributes of sesame plants treated with ginger-loaded chitosan nanoparticles and pesticides.

The control (untreated) plants showed moderate activity in most of the biochemical parameters (Fig 5a and b). Both concentrations of ChNPs improved enzymatic and pigment profiles, producing balanced polygons in the spider plot (Fig 6). ChNPs 50 mg L^-1^ increased PPO 2.6%, chlorophyll a 6.5%, but reduced catalase 28.5%, peroxidase −88.4%, and PAL 23%. These effects increased at ChNPs 100 mg L^-1^, catalase increased 53.1%, total chlorophyll increased 11.9%, and peroxidase was decreased 85.7%, PPO 18.2%, and PAL 59%. ChNPs penetrate the plant tissues effectively, release bioactive oligomers, and induce endogenous pathways, such as antioxidant enzyme upregulation and increased chlorophyll biosynthesis. Other crops have also been reported to have similar enhancement of antioxidant enzymes and pigments by ChNPs. Chitosan NPs at 20 mg L ^−1^ enhanced catalase, peroxidase, and other antioxidant enzymes, decreased hydrogen peroxide and MDA, and photosynthetic pigments in *Vicia faba* under drought, and thus, improved stress tolerance. Foliar ChNPs (300 mg L^-1^) in chamomile enhanced flower yield by approximately 52−56% and essential oil by 47−57%, along with chlorophyll and antioxidant capacity [40]. ChPs enhanced total chlorophyll by 63% and carotenoids by 68%, and SOD, PPO, and peroxidase activity by up to 73−86% in *Salvia abrotanoides* during drought stress [41]. Foliar ChNPs (20−100 ppm) increased chlorophyll content, total phenolics, flavonoids, and total antioxidant activity by 20−75%, and 82%, respectively, in *Capsicum annuum*. All these studies contribute to the opinion that ChNPs are powerful elicitors, which generally increase catalase, peroxidase, PPO, and PAL and enhance pigment status, but the percentage change and direction of each enzyme may depend on species, dose, and stress conditions. The asymmetric profiles of chemical pesticide treatments were observed in the same spider plot (Fig 6). The catalase activity of confidor (2.5 mL L^-1^) was near control levels, 0.9% increase, but reduced PPO by 15.2%, peroxidase by 84.4%, and PAL by 68%. The confidor + talstar (2.5 mL L^-1^) increased catalase 23.7%, PPO 6.3%, and peroxidase 38.3%, but decreased chlorophyll b 16% and total chlorophyll 4% showed non-significance (P > 0.05), which proves that these selective activations and inhibitions are due to actual treatment induced stress. The varietal responses in all treatments showed that there were inherent differences in biochemical profiles (Table 2). N- Millennium showed the growth of chlorophyll a 24.0, chlorophyll b 22.1, and total chlorophyll 18.9 over the overall control mean, moderate catalase 13.0, but reduced PAL 32.8. TH-6 had the best catalase 46.4% and peroxidase, 33.3%, high total chlorophyll, 14.8, and low PAL, 61.0. N- Pearl exhibited the least activity in peroxidase 82.6%, chlorophyll a 33.9% and chlorophyll b 44.5% but the increase in PPO 4.1%. Similar cultivar-dependent differences have been reported for common bean and other species treated with ChNPs, where some genotypes show larger gains in CAT and SOD (97% increase) and stronger pigment recovery under salinity. ChNPs, particularly at 100 mg L^-1^ demonstrated effects on defense enzymes and photosynthetic pigments, but chemical pesticides showed suppressive effects. This indicates the high potential of chitosan nanoparticles in plant biochemistry improvement, which provides a sustainable pathway to enhanced crop resilience and less chemical reliance.

**Table 2:** Effect of ginger-loaded ChNPs on biochemical attributes (Mean ± SE, n = 15).

Treatment	N-Millennium	TH-6	N-Pearl	TS-5	NS-2016
**Catalase treatment x variety interaction Mean ± SE (LSD = 0.0741)**					
**Control**	2.14 ± 0.03^i^	2.26 ± 0.02^g^	2.37 ± 0.03^h^	1.63 ± 0.02^K^	1.80 ± 0.01^j^
**ChNPs(50 mg L ⁻ ¹)**	1.57 ± 0.03^k^	2.76 ± 0.03^f^	1.01 ± 0.02^m^	1.22 ± 0.02^l^	0.85 ± 0.01^n^
**ChNPs(100 mg L ⁻ ¹)**	4.70 ± 0.05^b^	3.12 ± 0.03^e^	5.13 ± 0.04^a^	1.64 ± 0.02^k^	1.27 ± 0.01^l^
**Confidor (2.5 mL L ⁻ ¹)**	2.45 ± 0.03^g^	2.19 ± 0.03^i^	1.59 ± 0.02^k^	1.04 ± 0.01^m^	2.20 ± 0.01^i^
**Confidor+talstar (2.5 mL L ⁻ ¹)**	0.83 ± 0.02^n^	3.88 ± 0.03^d^	0.79 ± 0.02^n^	4.51 ± 0.04^c^	2.77 ± 0.01^f^
**Polyphenol oxidase treatment x variety interaction Mean ± SE (LSD = 2.2614)**					
**Control**	108.75 ± 0.74^l^	133.84 ± 1.32^f^	147.33 ± 0.87^b^	143.72 ± 0.80^c^	102.02 ± 0.17^m^
**ChNPs(50 mg L ⁻ ¹)**	117.82 ± 0.60^j^	127.94 ± 1.22^h^	134.51 ± 0.94^f^	129.12 ± 0.74^h^	142.78 ± 0.74^c^
**ChNPs(100 mg L ⁻ ¹)**	63.22 ± 0.45^p^	119.08 ± 1.33^j^	109.54 ± 0.74^l^	140.81 ± 0.74^d^	87.51 ± 0.17^o^
**Confidor (2.5 mL L ⁻ ¹)**	114.62 ± 0.43^k^	57.02 ± 0.70^q^	130.25 ± 0.90^g^	138.12 ± 0.74^e^	99.30 ± 0.12^n^
**Confidor+talstar (2.5 mL L ⁻ ¹)**	121.77 ± 0.48^j^	149.87 ± 1.42^a^	140.37 ± 0.87^d^	131.60 ± 0.79^g^	131.85 ± 0.79^g^
**Peroxidase treatment x variety interaction Mean ± SE (LSD = 0.5959)**					
**Control**	58.84 ± 0.55^b^	0.39 ± 0.00^h^	5.98 ± 0.02^c^	2.18 ± 0.01^f^	2.53 ± 0.023^e^
**ChNPs(50 mg L ⁻ ¹)**	0.95 ± 0.01^h^	2.13 ± 0.01^f^	1.58 ± 0.02^g^	2.84 ± 0.02^e^	3.97 ± 0.01^d^
**ChNPs(100 mg L ⁻ ¹)**	2.12 ± 0.02^f^	2.45 ± 0.01^e^	2.41 ± 0.02^e^	0.78 ± 0.01^h^	1.83 ± 0.02^f^
**Confidor (2.5 mL L ⁻ ¹)**	2.33 ± 0.02^e^	1.41 ± 0.02^g^	1.59 ± 0.02^f^	1.59 ± 0.02^a^	0.88 ± 0.02^h^
**Confidor+talstar (2.5 mL L ⁻ ¹)**	1.37 ± 0.02^g^	1.58 ± 0.89^a^	0.99 ± 0.02^h^	2.40 ± 0.02^e^	1.72 ± 0.02^f^
**Chlorophyll a treatment x variety interaction Mean ± SE (LSD = 0.9730)**					
**Control**	60.61 ± 0.39^j^	67.48 ± 0.35^g^	54.46 ± 0.32^n^	37.43 ± 0.33^q^	81.27 ± 0.17^b^
**ChNPs(50 mg L ⁻ ¹)**	89.50 ± 0.44^a^	51.66 ± 0.40^o^	45.18 ± 0.34^p^	64.30 ± 0.45ⁱ	70.22 ± 0.45^f^
**ChNPs(100 mg L ⁻ ¹)**	75.33 ± 0.43^d^	66.48 ± 0.33^h^	35.98 ± 0.27^r^	76.22 ± 0.47^d^	66.94 ± 0.47^g^
**Confidor (2.5 mL L ⁻ ¹)**	70.63 ± 0.34^f^	79.26 ± 0.46^c^	33.55 ± 0.27^s^	55.69 ± 0.44^m^	75.86 ± 0.44^d^
**Confidor+talstar (2.5 mL L ⁻ ¹)**	74.59 ± 0.26^e^	61.19 ± 0.49^j^	29.98 ± 0.25^t^	57.40 ± 0.40^l^	59.81 ± 0.40^k^
**Chlorophyll b treatment x variety interaction Mean ± SE (LSD = 1.1364)**					
**Control**	95.31 ± 0.39^f^	76.45 ± 0.34^l^	66.52 ± 0.32^o^	44.83 ± 0.38^q^	136.10 ± 0.12^a^
**ChNPs(50 mg L ⁻ ¹)**	125.62 ± 0.45^b^	71.70 ± 0.41^n^	51.04 ± 0.35^p^	85.86 ± 0.53^j^	90.48 ± 0.53^h^
**ChNPs(100 mg L ⁻ ¹)**	87.17 ± 0.42^j^	86.67 ± 0.41^i^	44.73 ± 0.30^q^	102.32 ± 0.82^d^	91.77 ± 0.82^g^
**Confidor (2.5 mL L ⁻ ¹)**	104.30 ± 0.45^c^	104.63 ± 0.45^c^	35.97 ± 0.26^r^	79.76 ± 0.68^k^	86.60 ± 0.68^i^
**Confidor+talstar (2.5 mL L ⁻ ¹)**	99.39 ± 0.34^e^	80.66 ± 0.45^k^	34.39 ± 0.27^s^	65.66 ± 0.45^o^	73.19 ± 0.45^m^
**Total Chlorophyll treatment x variety interaction Mean ± SE (LSD = 0.2928)**					
**Control**	17.09 ± 0.13^g^	18.04 ± 0.04^e^	14.19 ± 0.08^k^	14.65 ± 0.13^j^	16.69 ± 0.13^h^
**ChNPs(50 mg L ⁻ ¹)**	15.71 ± 0.13^i^	24.13 ± 0.04^a^	16.62 ± 0.08^h^	17.89 ± 0.14^e^	12.33 ± 0.01^m^
**ChNPs(100 mg L ⁻ ¹)**	24.40 ± 0.18^a^	19.16 ± 0.07^d^	14.56 ± 0.08^j^	19.28 ± 0.19^d^	12.87 ± 0.02l^i^
**Confidor (2.5 mL L ⁻ ¹)**	22.02 ± 0.15^b^	17.33 ± 0.05ᶠ	11.15 ± 0.08^n^	17.03 ± 0.13^g^	12.94 ± 0.01^l^
**Confidor+talstar (2.5 mL L ⁻ ¹)**	16.67 ± 0.15^h^	19.96 ± 0.07^c^	17.54 ± 0.09^f^	12.20 ± 0.14^m^	11.07 ± 0.02^n^
**Phenylalanine ammonia lyase treatment x variety interaction Mean ± SE (LSD = 0.0952)**					
**Control**	5.15 ± 0.04^d^	1.06 ± 0.01^m^	4.96 ± 0.03^e^	5.51 ± 0.06^c^	0.85 ± 0.01^o^
**ChNPs(50 mg L ⁻ ¹)**	3.07 ± 0.03^f^	0.60 ± 0.01^p^	1.70 ± 0.02^j^	7.64 ± 0.09^a^	0.47 ± 0.01^q^
**ChNPs(100 mg L ⁻ ¹)**	2.04 ± 0.03^i^	1.61 ± 0.03^k^	0.93 ± 0.02^n^	1.78 ± 0.03^j^	0.81 ± 0.01^o^
**Confidor (2.5 mL L ⁻ ¹)**	1.13 ± 0.02^m^	0.95 ± 0.02^n^	1.10 ± 0.02^m^	2.28 ± 0.04^h^	0.17 ± 0.01^r^
**Confidor+talstar (2.5 mL L ⁻ ¹)**	0.43 ± 0.02^q^	2.65 ± 0.03^g^	6.67 ± 0.05^b^	1.24 ± 0.03^l^	2.35 ± 0.03^h^

Means sharing similar letters in a row or in a column are statistically non-significant (P > 0.05).

Principal Component Analysis (PCA) biplot shows that ChNPs treatments, particularly at 100 mg L^-1^, significantly enhance plant morphological characteristics such as height, stem diameter, capsule weight, number of capsules, and leaf area, with strong positive relationships (Fig 6a). In contrast, the number of leaves has a negative correlation with other variables, and control or chemical treatments cluster away from growth related parameters. The first principal component (68.4% variance) reflects these relationships, indicating ChNPs’ significant impact on plant morphological parameters.

Multivariate correlation between enzyme activity and chlorophyll content across sesame plant samples (Fig 6b). Dim1 (35.9%) and Dim2 (20.2%) account for 56.1% of overall variance, indicating significant biochemical heterogeneity in sesame varieties. The vectors for chlorophyll a, chlorophyll b, and total chlorophyll correlate positively along Dim1, demonstrating that these parameters correlate strongly and identify samples on this axis. In contrast, enzymes such as polyphenol oxidase and phenylalanine ammonia lyase have a negative correlation with Dim1, indicating a negative correlation with chlorophyll concentration; however, Catalase and peroxidase vary independently. The clustering of ChNPs-treated samples near chlorophyll vectors demonstrates their ability to maintain photosynthetic integrity, as compared to insecticide-treated sesame plants, which cluster further away and indicate metabolic suppression [42]. These findings support the significance of chitosan in modifying plant stress Table 3 responses [42].

**Table 3:** PCA biplot loading table of the effect of different treatments on morphological and biochemical attributes of *S. indicum* L.

	Morphological atrributes					Biochemical attributes				
Treatment	PC1	PC2	PC3	PC4	PC5	PC1	PC2	PC3	PC4	PC5
**Control**	0.1508	−1.9113	0.0520	0.3552	0.1143	0.1448	0.6575	−0.2014	−0.0345	−0.0938
**ChNPs (50 mg L ⁻ ¹)**	2.6171	1.4405	−0.5092	0.1237	1.50515	−0.4404	−0.2913	0.0730	0.4098	−0.6640
**ChNPs (100 mg L ⁻ ¹)**	1.0933	−0.3553	1.1024	−0.3171	−6.2215	−0.4381	0.2653	−0.2313	−0.4617	−0.1861
**Confidor (2.5 mL L ⁻ ¹)**	−2.6828	1.4090	0.4260	0.1473	−2.1115	0.4355	−0.3166	0.0682	0.1506	0.1722
**Confidor + talstar (2.5 mL L ⁻ ¹)**	−1.1785	−0.5829	−1.0712	−0.3092	2.7815	0.3187	−0.4571	−0.4141	−0.5702	−0.3546

## Conclusion

This study effectively demonstrated the synthesis of ginger-loaded chitosan nanoparticles (ChNPs) loaded with ginger extract, which have stable, monodisperse structures (151.7 nm, + 31.4 mV zeta potential), a semi-crystalline morphology, and bioactive functional groups. ChNPs application, particularly at 100 mg L^-1^, significantly improved sesame plants’ morphological traits such as plant height, capsule weight, and leaf area while maintaining higher enzymatic activities and chlorophyll content than chemical pesticides such as confidor and confidor+talstar. The dose-dependent response and varietal differences, with N- Millennium and TH-6, showed better morphological and biochemical parameters. Significant treatment effects and trait correlations highlight the effectiveness of ChNPs in improving plant physiology and growth. These findings establish ChNPs as a sustainable alternative to traditional chemical pesticides. Future studies on investigating long-term field efficacy and monitoring environmental implications of ginger-loaded ChNPs may contribute to precision agriculture and sustainable crop management.

## Supporting information

S1 File Supporting information. (DOCX)
