# Supplementary material for: Effects of ginger-loaded chitosan nanoparticles on growth, morphological and biochemical attributes of Sesamum indicum L
Source: PLoS One. 2026 Jul 2;21(7):e0349701. doi: 10.1371/journal.pone.0349701 (PMC13327131; doi:10.1371/journal.pone.0349701)
Supplement: S1 File — (DOCX) [file pone.0349701.s001.docx]

Fig 2. (a) UV- vis spectrophotometer analysis of ginger loaded chitosan nanoparticles

| Wavelength (cm^-1)^ | Absorbance |
| --- | --- |
| 200 | 0.1 |
| 210 | 0.1 |
| 220 | 0.11 |
| 230 | 0.12 |
| 240 | 0.14 |
| 250 | 0.2 |
| 263 | 0.734 |
| 264 | 0.82 |
| 265 | 0.912 |
| 266 | 1.006 |
| 267 | 1.098 |
| 268 | 1.181 |
| 269 | 1.251 |
| 270 | 1.303 |
| 271 | 1.335 |
| 272 | 1.349 |
| 273 | 1.345 |
| 274 | 1.328 |
| 275 | 1.302 |
| 276 | 1.271 |
| 277 | 1.243 |
| 278 | 1.22 |
| 279 | 1.209 |
| 280 | 1.214 |
| 281 | 1.238 |
| 282 | 1.282 |
| 283 | 1.344 |
| 284 | 1.422 |
| 285 | 1.511 |
| 286 | 1.604 |
| 287 | 1.696 |
| 288 | 1.783 |
| 289 | 1.859 |
| 290 | 1.925 |
| 291 | 1.979 |
| 292 | 2.021 |
| 293 | 2.053 |
| 294 | 2.074 |
| 295 | 2.086 |
| 296 | 2.09 |
| 297 | 2.085 |
| 298 | 2.072 |
| 299 | 2.052 |
| 300 | 2.024 |
| 301 | 1.989 |
| 302 | 1.948 |
| 303 | 1.9 |
| 304 | 1.847 |
| 305 | 1.79 |
| 306 | 1.728 |
| 307 | 1.662 |
| 308 | 1.594 |
| 309 | 1.523 |
| 310 | 1.451 |
| 311 | 1.378 |
| 312 | 1.305 |
| 313 | 1.233 |
| 314 | 1.161 |
| 315 | 1.091 |
| 316 | 1.023 |
| 317 | 0.957 |
| 318 | 0.894 |
| 319 | 0.833 |
| 320 | 0.776 |
| 321 | 0.722 |
| 322 | 0.672 |
| 323 | 0.625 |
| 324 | 0.581 |
| 325 | 0.541 |
| 326 | 0.504 |
| 327 | 0.47 |
| 328 | 0.44 |
| 329 | 0.412 |
| 330 | 0.387 |
| 331 | 0.364 |
|  |  |
| 332 | 0.344 |
| 333 | 0.326 |
| 334 | 0.31 |
| 335 | 0.296 |
| 336 | 0.284 |
| 337 | 0.273 |
| 338 | 0.263 |
| 339 | 0.255 |
| 340 | 0.247 |
| 341 | 0.241 |
| 342 | 0.235 |
| 343 | 0.23 |
| 344 | 0.225 |
| 345 | 0.221 |
| 346 | 0.217 |
| 347 | 0.214 |
| 348 | 0.211 |
| 349 | 0.208 |
| 350 | 0.206 |
| 351 | 0.203 |
| 352 | 0.201 |
| 353 | 0.199 |
| 354 | 0.197 |
| 355 | 0.195 |
| 356 | 0.193 |
| 357 | 0.191 |
| 358 | 0.189 |
| 359 | 0.188 |
| 360 | 0.186 |
| 361 | 0.184 |
| 362 | 0.183 |
| 363 | 0.181 |
| 364 | 0.179 |
| 365 | 0.178 |
| 366 | 0.176 |
| 367 | 0.174 |
| 368 | 0.173 |
| 369 | 0.171 |
| 370 | 0.169 |
| 371 | 0.168 |
| 372 | 0.166 |
| 373 | 0.164 |
| 374 | 0.163 |
| 375 | 0.161 |
| 376 | 0.159 |
| 377 | 0.158 |
| 378 | 0.156 |
| 379 | 0.155 |
| 380 | 0.153 |
| 381 | 0.151 |
| 382 | 0.15 |
| 383 | 0.148 |
| 384 | 0.146 |
| 385 | 0.145 |
| 386 | 0.143 |
| 387 | 0.141 |
| 388 | 0.14 |
| 389 | 0.138 |
| 390 | 0.136 |
| 391 | 0.135 |
| 392 | 0.133 |
| 393 | 0.132 |
| 394 | 0.13 |
| 395 | 0.128 |
| 396 | 0.127 |
| 397 | 0.125 |
| 398 | 0.123 |
| 399 | 0.122 |
| 400 | 0.12 |
| 401 | 0.09269 |
| 404 | 0.08429 |
| 407 | 0.07728 |
| 410 | 0.06147 |
| 413 | 0.05671 |
| 416 | 0.04284 |
| 419 | 0.03972 |
| 422 | 0.02724 |
| 425 | 0.01529 |
| 428 | 0.01378 |
| 431 | 0.01263 |
| 434 | 0.01178 |
| 437 | 0.01118 |
| 440 | 0.01077 |
| 443 | 0.01052 |
| 446 | 0.0104 |
| 449 | 0.01037 |
| 452 | 0.01042 |
| 455 | 0.01054 |
| 458 | 0.0107 |
| 461 | 0.0109 |
| 464 | 0.01112 |
| 467 | 0.01137 |
| 470 | 0.01164 |
| 473 | 0.01191 |
| 476 | 0.0122 |
| 479 | 0.01249 |
| 482 | 0.01278 |
| 485 | 0.01308 |
| 488 | 0.01338 |
| 491 | 0.01368 |
| 494 | 0.01399 |
| 497 | 0.01429 |
| 500 | 0.01459 |
| 503 | 0.01489 |
| 506 | 0.01519 |
| 509 | 0.01548 |
| 512 | 0.01578 |
| 515 | 0.01607 |
| 518 | 0.01637 |
| 521 | 0.01665 |
| 524 | 0.01694 |
| 527 | 0.01723 |
| 530 | 0.01751 |
| 533 | 0.01779 |
| 536 | 0.01806 |
| 539 | 0.01834 |
| 542 | 0.01861 |
| 545 | 0.01887 |
| 548 | 0.01914 |
| 551 | 0.0194 |
| 554 | 0.01966 |
| 557 | 0.01991 |
| 560 | 0.02016 |
| 563 | 0.02041 |
| 566 | 0.02066 |
| 569 | 0.0209 |
| 572 | 0.02113 |
| 575 | 0.02137 |
| 578 | 0.0216 |
| 581 | 0.02182 |
| 584 | 0.02204 |
| 587 | 0.02226 |
| 590 | 0.02247 |
| 593 | 0.02269 |
| 596 | 0.02289 |
| 599 | 0.0231 |
| 602 | 0.02329 |
| 605 | 0.02349 |
| 608 | 0.02368 |
| 611 | 0.02387 |
| 614 | 0.02405 |
| 617 | 0.02423 |
| 620 | 0.02441 |
| 623 | 0.02458 |
| 626 | 0.02475 |
| 629 | 0.02491 |
| 632 | 0.02507 |
| 635 | 0.02523 |
| 638 | 0.02538 |
| 641 | 0.02553 |
| 644 | 0.02568 |
| 647 | 0.02582 |
| 650 | 0.02596 |
| 653 | 0.0261 |
| 656 | 0.02623 |
| 659 | 0.02636 |
| 662 | 0.02648 |
| 665 | 0.0266 |
| 668 | 0.02672 |
| 671 | 0.02684 |
| 674 | 0.02695 |
| 677 | 0.02706 |
| 680 | 0.02717 |
| 683 | 0.02727 |
| 686 | 0.02737 |
| 689 | 0.02747 |
| 692 | 0.02756 |
| 695 | 0.02765 |
| 698 | 0.02774 |
| 701 | 0.02783 |
| 704 | 0.02791 |
| 707 | 0.02799 |
| 710 | 0.02807 |
| 713 | 0.02814 |
| 716 | 0.02822 |
| 719 | 0.02829 |
| 722 | 0.02836 |
| 725 | 0.02842 |
| 728 | 0.02849 |
| 731 | 0.02855 |
| 734 | 0.02861 |
| 737 | 0.02866 |
| 740 | 0.02872 |
| 743 | 0.02877 |
| 746 | 0.02883 |
| 749 | 0.02888 |
| 752 | 0.02892 |
| 755 | 0.02897 |
| 758 | 0.02901 |
| 761 | 0.02906 |
| 764 | 0.0291 |
| 767 | 0.02914 |
| 770 | 0.02918 |
| 773 | 0.02921 |
| 776 | 0.02925 |
| 779 | 0.02928 |
| 782 | 0.02932 |
| 785 | 0.02935 |
| 788 | 0.02938 |
| 791 | 0.02941 |
| 794 | 0.02943 |
| 797 | 0.02946 |
| 800 | 0.02949 |

**Fig 2. (b) FTIR analysis of ginger loaded chitosan nanoparticles**

| **Wavenumber (cm^-1^)** | **Transmittance** |
| --- | --- |
| 4000 | 89.9 |
| 4000 | 89.9 |
| 3982 | 89.19 |
| 3965 | 89.78 |
| 3947 | 89.37 |
| 3930 | 89.16 |
| 3912 | 89.44 |
| 3894 | 89.91 |
| 3877 | 88.98 |
| 3859 | 88.34 |
| 3842 | 87.8 |
| 3824 | 87.81 |
| 3807 | 86.2 |
| 3789 | 85 |
| 3771 | 85 |
| 3754 | 83.7 |
| 3736 | 82 |
| 3719 | 80.1 |
| 3701 | 77.8 |
| 3683 | 75.3 |
| 3666 | 72.4 |
| 3648 | 69.3 |
| 3631 | 66 |
| 3613 | 62.5 |
| 3500 | 8.41 |
| 3485 | 6.21 |
| 3470 | 4.28 |
| 3455 | 2.64 |
| 3440 | 1.36 |
| 3425 | 0.46 |
| 3410 | 0 |
| 3394 | 0 |
| 3379 | 0.28 |
| 3364 | 1.06 |
| 3349 | 2.23 |
| 3334 | 3.77 |
| 3319 | 5.62 |
| 3304 | 7.74 |
| 3289 | 10.08 |
| 3274 | 12.6 |
| 3259 | 15.23 |
| 3244 | 17.95 |
| 3229 | 20.7 |
| 3214 | 23.45 |
| 3198 | 26.18 |
| 3183 | 28.85 |
| 3168 | 31.45 |
| 3153 | 33.96 |
| 3138 | 36.38 |
| 3123 | 38.68 |
| 3108 | 40.86 |
| 3093 | 42.92 |
| 3078 | 44.85 |
| 3063 | 46.64 |
| 3048 | 48.27 |
| 3033 | 49.73 |
| 3018 | 50.94 |
| 3003 | 51.83 |
| 2987 | 52.13 |
| 2972 | 51.3 |
| 2957 | 47.74 |
| 2942 | 45.07 |
| 2927 | 44.08 |
| 2912 | 43.41 |
| 2897 | 47.73 |
| 2882 | 51.36 |
| 2867 | 60.78 |
| 2852 | 62.91 |
| 2837 | 64.43 |
| 2822 | 65.63 |
| 2807 | 66.64 |
| 2791 | 67.52 |
| 2776 | 68.31 |
| 2761 | 69.02 |
| 2746 | 69.99 |
| 2731 | 70 |
| 2716 | 70.36 |
| 2701 | 71.99 |
| 2686 | 71 |
| 2671 | 72.36 |
| 2656 | 72.81 |
| 2641 | 73.23 |
| 2626 | 73.23 |
| 2611 | 74.42 |
| 2595 | 74.56 |
| 2580 | 74.71 |
| 2565 | 75.43 |
| 2550 | 75.34 |
| 2535 | 75.84 |
| 2520 | 75.92 |
| 2505 | 76.28 |
| 2490 | 76.14 |
| 2475 | 76.99 |
| 2460 | 76.92 |
| 2445 | 77.24 |
| 2430 | 77.36 |
| 2415 | 77.66 |
| 2399 | 77.76 |
| 2384 | 77.99 |
| 2369 | 78.12 |
| 2354 | 78.59 |
| 2339 | 78.06 |
| 2324 | 78.62 |
| 2309 | 78.37 |
| 2294 | 78.91 |
| 2279 | 79.95 |
| 2264 | 79.18 |
| 2249 | 79.61 |
| 2234 | 79.43 |
| 2219 | 79.55 |
| 2204 | 79.46 |
| 2188 | 79.77 |
| 2173 | 79.97 |
| 2158 | 79.97 |
| 2143 | 80.06 |
| 2128 | 80.55 |
| 2113 | 80.63 |
| 2098 | 80.31 |
| 2083 | 80.48 |
| 2068 | 80.75 |
| 2053 | 80.52 |
| 2038 | 80.54 |
| 2023 | 80.26 |
| 2008 | 80.65 |
| 1992 | 80.72 |
| 1977 | 80.88 |
| 1962 | 80.75 |
| 1947 | 80.81 |
| 1932 | 80.88 |
| 1917 | 80.8 |
| 1902 | 80.81 |
| 1887 | 80.72 |
| 1872 | 80.74 |
| 1857 | 80.67 |
| 1842 | 80.58 |
| 1827 | 80.45 |
| 1812 | 80.27 |
| 1796 | 80.02 |
| 1781 | 79.67 |
| 1766 | 79.19 |
| 1751 | 78.48 |
| 1736 | 77.42 |
| 1721 | 75.75 |
| 1706 | 72.9 |
| 1691 | 67.6 |
| 1676 | 50.43 |
| 1661 | 44.17 |
| 1646 | 35.23 |
| 1631 | 37.78 |
| 1616 | 40.35 |
| 1601 | 46.58 |
| 1585 | 53.34 |
| 1570 | 73.6 |
| 1555 | 74.89 |
| 1540 | 76.18 |
| 1525 | 75.9 |
| 1510 | 77.22 |
| 1495 | 76.22 |
| 1480 | 77.92 |
| 1465 | 77.25 |
| 1450 | 76.15 |
| 1435 | 74.56 |
| 1420 | 73.75 |
| 1405 | 71.57 |
| 1389 | 72.96 |
| 1374 | 73.64 |
| 1359 | 75.54 |
| 1344 | 77.08 |
| 1329 | 78.16 |
| 1314 | 78.88 |
| 1299 | 79.34 |
| 1284 | 79.61 |
| 1269 | 79.73 |
| 1254 | 79.71 |
| 1239 | 79.57 |
| 1224 | 79.3 |
| 1209 | 78.86 |
| 1193 | 78.19 |
| 1178 | 77.18 |
| 1163 | 75.62 |
| 1148 | 67.07 |
| 1133 | 56.55 |
| 1118 | 48.5 |
| 1103 | 23.07 |
| 1088 | 28.47 |
| 1073 | 30.67 |
| 1058 | 33.97 |
| 1043 | 40.72 |
| 1028 | 42.21 |
| 1013 | 43.8 |
| 997 | 45.71 |
| 982 | 57.23 |
| 967 | 63.45 |
| 952 | 65.89 |
| 937 | 65.83 |
| 922 | 63.89 |
| 907 | 60.78 |
| 892 | 58.19 |
| 877 | 58.19 |
| 862 | 60.64 |
| 847 | 63.41 |
| 832 | 65.09 |
| 817 | 65.78 |
| 802 | 66.6 |
| 786 | 68.54 |
| 771 | 71.22 |
| 756 | 73.7 |
| 741 | 75.6 |
| 726 | 76.93 |
| 711 | 77.81 |
| 696 | 78.36 |
| 681 | 78.64 |
| 666 | 78.68 |
| 651 | 78.48 |
| 636 | 77.99 |
| 621 | 77.14 |
| 606 | 75.78 |
| 590 | 73.73 |
| 575 | 70.81 |
| 560 | 67.14 |
| 545 | 63.88 |
| 530 | 63.14 |
| 515 | 65.62 |
| 500 | 69.52 |

Fig 2. (d) XRD analysis of ginger loaded chitosan nanoparticles

| **Angle [°2Theta]** | **Intensity [counts]** |
| --- | --- |
| 10.0001 | 0.38747 |
| 10.02042 | 0.76921 |
| 10.04074 | 1.05239 |
| 10.06106 | 1.24415 |
| 10.08138 | 1.35161 |
| 10.10171 | 1.38191 |
| 10.12203 | 1.34217 |
| 10.14235 | 1.23953 |
| 10.16267 | 1.0811 |
| 10.18299 | 0.87402 |
| 10.20331 | 0.62543 |
| 10.22363 | 0.34244 |
| 10.24395 | 0.03218 |
| 10.26428 | 0.29821 |
| 10.2846 | 0.6416 |
| 10.30492 | 0.99088 |
| 10.32524 | 1.3389 |
| 10.34556 | 1.67854 |
| 10.36588 | 2.00268 |
| 10.3862 | 2.30417 |
| 10.40652 | 2.5759 |
| 10.42685 | 2.81074 |
| 10.44717 | 3.00155 |
| 10.46749 | 3.14121 |
| 10.48781 | 3.22259 |
| 10.50813 | 3.23855 |
| 10.52845 | 2.50669 |
| 10.54877 | 6.70549 |
| 10.56909 | 2.32767 |
| 10.58942 | 2.24799 |
| 10.60974 | 6.82114 |
| 10.63006 | 3.12129 |
| 10.65038 | 15.69633 |
| 10.6707 | 14.05826 |
| 10.69102 | 7.08916 |
| 10.71134 | 8.07817 |
| 10.73166 | 4.24708 |
| 10.75198 | 3.44468 |
| 10.77231 | 12.76138 |
| 10.79263 | 19.48443 |
| 10.81295 | 23.33895 |
| 10.83327 | 19.60741 |
| 10.85359 | 25.24022 |
| 10.87391 | 24.78959 |
| 10.89423 | 24.73322 |
| 10.91455 | 22.28204 |
| 10.93488 | 28.79314 |
| 10.9552 | 31.21425 |
| 10.97552 | 20.0524 |
| 10.99584 | 18.41194 |
| 11.01616 | 17.66991 |
| 11.03648 | 14.72488 |
| 11.0568 | 15.57053 |
| 11.07712 | 22.28265 |
| 11.09745 | 21.17906 |
| 11.11777 | 18.56033 |
| 11.13809 | 17.69371 |
| 11.15841 | 15.66846 |
| 11.17873 | 2.20859 |
| 11.19905 | 6.39521 |
| 11.21937 | 3.92802 |
| 11.23969 | 5.78082 |
| 11.26002 | 18.33539 |
| 11.28034 | 34.18599 |
| 11.30066 | 41.73583 |
| 11.32098 | 50.80537 |
| 11.3413 | 47.78307 |
| 11.36162 | 54.41581 |
| 11.38194 | 59.26763 |
| 11.40226 | 64.15587 |
| 11.42258 | 69.21946 |
| 11.44291 | 77.81846 |
| 11.46323 | 82.56254 |
| 11.48355 | 90.51738 |
| 11.50387 | 93.20496 |
| 11.52419 | 92.65361 |
| 11.54451 | 106.6098 |
| 11.56483 | 113.63891 |
| 11.58515 | 114.0697 |
| 11.60548 | 110.6468 |
| 11.6258 | 112.80245 |
| 11.64612 | 118.76388 |
| 11.66644 | 123.18541 |
| 11.68676 | 121.08081 |
| 11.70708 | 123.98407 |
| 11.7274 | 131.03251 |
| 11.74772 | 129.11696 |
| 11.76805 | 120.38865 |
| 11.78837 | 115.47664 |
| 11.80869 | 120.37323 |
| 11.82901 | 122.64765 |
| 11.84933 | 123.31925 |
| 11.86965 | 121.77768 |
| 11.88997 | 125.24878 |
| 11.91029 | 123.80398 |
| 11.93061 | 123.75449 |
| 11.95094 | 115.97561 |
| 11.97126 | 103.06296 |
| 11.99158 | 102.23806 |
| 12.0119 | 101.70979 |
| 12.03222 | 93.53061 |
| 12.05254 | 91.95754 |
| 12.07286 | 90.86549 |
| 12.09318 | 87.93717 |
| 12.11351 | 92.11285 |
| 12.13383 | 84.70655 |
| 12.15415 | 81.03264 |
| 12.17447 | 75.51509 |
| 12.19479 | 83.0961 |
| 12.21511 | 72.79838 |
| 12.23543 | 76.04515 |
| 12.25575 | 69.60879 |
| 12.27608 | 71.17086 |
| 12.2964 | 58.569 |
| 12.31672 | 52.04248 |
| 12.33704 | 44.53957 |
| 12.35736 | 36.35754 |
| 12.37768 | 35.25814 |
| 12.398 | 34.11603 |
| 12.41832 | 32.02709 |
| 12.43865 | 31.88409 |
| 12.45897 | 31.93782 |
| 12.47929 | 29.52443 |
| 12.49961 | 26.93941 |
| 12.51993 | 31.29307 |
| 12.54025 | 35.56463 |
| 12.56057 | 36.60402 |
| 12.58089 | 28.93035 |
| 12.60121 | 29.71781 |
| 12.62154 | 35.45746 |
| 12.64186 | 41.64645 |
| 12.66218 | 40.93317 |
| 12.6825 | 36.24958 |
| 12.70282 | 34.4231 |
| 12.72314 | 29.0958 |
| 12.74346 | 22.00325 |
| 12.76378 | 11.26892 |
| 12.78411 | 14.64892 |
| 12.80443 | 19.55322 |
| 12.82475 | 27.39621 |
| 12.84507 | 21.2247 |
| 12.86539 | 16.09581 |
| 12.88571 | 13.02785 |
| 12.90603 | 7.64384 |
| 12.92635 | 5.59554 |
| 12.94668 | 4.01104 |
| 12.967 | 8.2608 |
| 12.98732 | 6.43352 |
| 13.00764 | 9.75799 |
| 13.02796 | 13.85515 |
| 13.04828 | 13.41841 |
| 13.0686 | 22.59529 |
| 13.08892 | 30.32414 |
| 13.10925 | 37.93249 |
| 13.12957 | 45.70096 |
| 13.14989 | 45.60852 |
| 13.17021 | 48.55822 |
| 13.19053 | 50.79911 |
| 13.21085 | 48.14784 |
| 13.23117 | 42.10476 |
| 13.25149 | 43.80418 |
| 13.27181 | 43.87058 |
| 13.29214 | 43.88539 |
| 13.31246 | 40.04714 |
| 13.33278 | 49.07466 |
| 13.3531 | 45.98254 |
| 13.37342 | 47.27758 |
| 13.39374 | 52.39849 |
| 13.41406 | 61.53348 |
| 13.43438 | 63.07002 |
| 13.45471 | 59.682 |
| 13.47503 | 60.02305 |
| 13.49535 | 56.39921 |
| 13.51567 | 58.8553 |
| 13.53599 | 62.18621 |
| 13.55631 | 52.8206 |
| 13.57663 | 48.71876 |
| 13.59695 | 44.27964 |
| 13.61728 | 46.1428 |
| 13.6376 | 41.87213 |
| 13.65792 | 39.76022 |
| 13.67824 | 35.73289 |
| 13.69856 | 26.90857 |
| 13.71888 | 27.73604 |
| 13.7392 | 19.66648 |
| 13.75952 | 15.12941 |
| 13.77985 | 13.75406 |
| 13.80017 | 35.31846 |
| 13.82049 | 45.26216 |
| 13.84081 | 63.78721 |
| 13.86113 | 76.02127 |
| 13.88145 | 96.26161 |
| 13.90177 | 116.79504 |
| 13.92209 | 136.28565 |
| 13.94241 | 160.33041 |
| 13.96274 | 169.3359 |
| 13.98306 | 192.6754 |
| 14.00338 | 208.84266 |
| 14.0237 | 224.62447 |
| 14.04402 | 244.74962 |
| 14.06434 | 258.81855 |
| 14.08466 | 282.02529 |
| 14.10498 | 295.05366 |
| 14.12531 | 297.25792 |
| 14.14563 | 302.04018 |
| 14.16595 | 302.63949 |
| 14.18627 | 509.4355 |
| 14.20659 | 313.28937 |
| 14.22691 | 310.81579 |
| 14.24723 | 619.2208 |
| 14.26755 | 318.93433 |
| 14.28788 | 319.33033 |
| 14.3082 | 320.40836 |
| 14.32852 | 321.02979 |
| 14.34884 | 315.99043 |
| 14.36916 | 311.77018 |
| 14.38948 | 308.13597 |
| 14.4098 | 299.634 |
| 14.43012 | 289.54886 |
| 14.45044 | 277.61373 |
| 14.47077 | 264.11085 |
| 14.49109 | 258.68416 |
| 14.51141 | 244.63296 |
| 14.53173 | 230.65145 |
| 14.55205 | 205.21504 |
| 14.57237 | 184.45933 |
| 14.59269 | 178.65795 |
| 14.61301 | 160.31108 |
| 14.63334 | 147.20374 |
| 14.65366 | 129.42109 |
| 14.67398 | 115.79258 |
| 14.6943 | 104.31931 |
| 14.71462 | 94.58338 |
| 14.73494 | 92.25763 |
| 14.75526 | 82.76736 |
| 14.77558 | 83.59858 |
| 14.79591 | 93.80678 |
| 14.81623 | 104.20557 |
| 14.83655 | 100.30564 |
| 14.85687 | 101.70222 |
| 14.87719 | 110.11881 |
| 14.89751 | 115.16039 |
| 14.91783 | 109.63882 |
| 14.93815 | 107.07198 |
| 14.95848 | 104.54995 |
| 14.9788 | 96.94331 |
| 14.99912 | 99.3131 |
| 15.01944 | 92.32571 |
| 15.03976 | 83.49074 |
| 15.0804 | 85.50743 |
| 15.10072 | 77.23613 |
| 15.12104 | 77.48718 |
| 15.14137 | 71.22628 |
| 15.16169 | 62.10605 |
| 15.18201 | 69.90863 |
| 15.20233 | 64.33866 |
| 15.22265 | 64.4455 |
| 15.24297 | 60.80201 |
| 15.26329 | 64.29496 |
| 15.28361 | 63.76239 |
| 15.30394 | 61.79849 |
| 15.32426 | 64.28176 |
| 15.34458 | 61.60567 |
| 15.3649 | 58.09694 |
| 15.38522 | 50.83863 |
| 15.40554 | 53.51036 |
| 15.42586 | 55.0643 |
| 15.44618 | 59.23919 |
| 15.46651 | 61.86302 |
| 15.48683 | 56.06634 |
| 15.50715 | 58.25814 |
| 15.52747 | 56.00101 |
| 15.54779 | 49.69293 |
| 15.56811 | 47.79621 |
| 15.58843 | 46.70457 |
| 15.60875 | 50.1364 |
| 15.62908 | 46.61013 |
| 15.6494 | 36.54729 |
| 15.66972 | 28.10823 |
| 15.69004 | 27.3667 |
| 15.71036 | 24.49555 |
| 15.73068 | 24.81404 |
| 15.751 | 17.31401 |
| 15.77132 | 19.66495 |
| 15.79164 | 16.61716 |
| 15.81197 | 13.49104 |
| 15.83229 | 15.92772 |
| 15.85261 | 11.69388 |
| 15.87293 | 13.46319 |
| 15.89325 | 13.13176 |
| 15.91357 | 17.3686 |
| 15.93389 | 13.76641 |
| 15.95421 | 7.13213 |
| 15.97454 | 9.7684 |
| 15.99486 | 10.61884 |
| 16.01518 | 5.65459 |
| 16.0355 | 4.43012 |
| 16.05582 | 7.74026 |
| 16.07614 | 7.21926 |
| 16.09646 | 8.45322 |
| 16.11678 | 11.43059 |
| 16.13711 | 15.5584 |
| 16.15743 | 13.61815 |
| 16.17775 | 17.5791 |
| 16.19807 | 22.69187 |
| 16.21839 | 20.12861 |
| 16.23871 | 22.61768 |
| 16.25903 | 24.54974 |
| 16.27935 | 24.57061 |
| 16.29968 | 27.96951 |
| 16.32 | 33.17706 |
| 16.34032 | 36.54752 |
| 16.36064 | 34.181 |
| 16.38096 | 34.8463 |
| 16.40128 | 35.3269 |
| 16.4216 | 41.5032 |
| 16.44192 | 44.25684 |
| 16.46224 | 47.3991 |
| 16.48257 | 45.6466 |
| 16.50289 | 43.5259 |
| 16.52321 | 46.12102 |
| 16.54353 | 34.26151 |
| 16.56385 | 32.79082 |
| 16.58417 | 33.45041 |
| 16.60449 | 25.41382 |
| 16.62481 | 18.49253 |
| 16.64514 | 10.85257 |
| 16.66546 | 6.69389 |
| 16.68578 | 4.70648 |
| 16.7061 | 14.64013 |
| 16.72642 | 12.41035 |
| 16.74674 | 10.22928 |
| 16.76706 | 7.6175 |
| 16.78738 | 14.17553 |
| 16.80771 | 4.05328 |
| 16.82803 | 0.8321 |
| 16.84835 | -1.57411 |
| 16.86867 | -0.58629 |
| 16.88899 | 0.55233 |
| 16.90931 | -0.18404 |
| 16.92963 | 2.91014 |
| 16.94995 | 6.42128 |
| 16.97027 | 5.05106 |
| 16.9906 | 3.84996 |
| 17.01092 | 3.35533 |
| 17.03124 | -3.60051 |
| 17.05156 | -0.34869 |
| 17.07188 | -2.29673 |
| 17.0922 | -5.48883 |
| 17.11252 | -4.66161 |
| 17.13284 | -1.09066 |
| 17.15317 | 2.05372 |
| 17.17349 | 1.61909 |
| 17.19381 | 3.55087 |
| 17.21413 | -1.58527 |
| 17.23445 | 0.99026 |
| 17.25477 | 3.56139 |
| 17.27509 | 4.3395 |
| 17.29541 | 3.8821 |
| 17.31574 | 9.56567 |
| 17.33606 | 9.04958 |
| 17.35638 | 5.73218 |
| 17.3767 | 5.07031 |
| 17.39702 | 2.80893 |
| 17.41734 | 3.77018 |
| 17.43766 | -0.18146 |
| 17.45798 | -1.32593 |
| 17.47831 | -10.72906 |
| 17.49863 | -17.45689 |
| 17.51895 | -18.47892 |
| 17.53927 | -18.74578 |
| 17.55959 | -23.86252 |
| 17.57991 | -12.1257 |
| 17.60023 | -16.5308 |
| 17.62055 | -23.22871 |
| 17.64087 | -16.70166 |
| 17.6612 | -15.32778 |
| 17.68152 | -9.81007 |
| 17.70184 | -0.58582 |
| 17.72216 | 3.75641 |
| 17.74248 | 4.22149 |
| 17.7628 | 6.02322 |
| 17.78312 | 8.53833 |
| 17.80344 | 23.47794 |
| 17.82377 | 22.14771 |
| 17.84409 | 24.07184 |
| 17.86441 | 34.27137 |
| 17.88473 | 38.95717 |
| 17.90505 | 38.02718 |
| 17.92537 | 45.77353 |
| 17.94569 | 49.39444 |
| 17.96601 | 47.91579 |
| 17.98634 | 44.14499 |
| 18.00666 | 45.30424 |
| 18.02698 | 48.76437 |
| 18.0473 | 53.60561 |
| 18.06762 | 59.10687 |
| 18.08794 | 62.52886 |
| 18.10826 | 68.24006 |
| 18.12858 | 63.94334 |
| 18.14891 | 67.42378 |
| 18.16923 | 74.16314 |
| 18.18955 | 69.59197 |
| 18.20987 | 72.41159 |
| 18.23019 | 70.60548 |
| 18.25051 | 79.08825 |
| 18.27083 | 78.54696 |
| 18.29115 | 84.51093 |
| 18.31147 | 86.68669 |
| 18.3318 | 90.80425 |
| 18.35212 | 89.11154 |
| 18.37244 | 85.66105 |
| 18.39276 | 82.62326 |
| 18.41308 | 81.0567 |
| 18.4334 | 79.97775 |
| 18.45372 | 80.47441 |
| 18.47404 | 79.84341 |
| 18.49437 | 77.45017 |
| 18.51469 | 84.77228 |
| 18.53501 | 86.14129 |
| 18.55533 | 79.73504 |
| 18.57565 | 79.28962 |
| 18.59597 | 83.84856 |
| 18.61629 | 72.38386 |
| 18.63661 | 75.97557 |
| 18.65694 | 79.57407 |
| 18.67726 | 76.15165 |
| 18.69758 | 79.88424 |
| 18.7179 | 79.6247 |
| 18.73822 | 78.58994 |
| 18.75854 | 76.20766 |
| 18.77886 | 72.24533 |
| 18.79918 | 67.10736 |
| 18.81951 | 71.98909 |
| 18.83983 | 60.91312 |
| 18.86015 | 58.15961 |
| 18.88047 | 52.5995 |
| 18.90079 | 48.0769 |
| 18.92111 | 44.47186 |
| 18.94143 | 46.93973 |
| 18.96175 | 39.49496 |
| 18.98207 | 37.0861 |
| 19.0024 | 35.19468 |
| 19.02272 | 30.77429 |
| 19.04304 | 33.39464 |
| 19.06336 | 34.35575 |
| 19.08368 | 31.60216 |
| 19.104 | 34.14684 |
| 19.12432 | 29.64147 |
| 19.14464 | 26.29992 |
| 19.16497 | 30.55457 |
| 19.18529 | 30.12687 |
| 19.20561 | 25.8629 |
| 19.22593 | 29.63278 |
| 19.24625 | 30.10496 |
| 19.26657 | 34.51505 |
| 19.28689 | 28.0109 |
| 19.30721 | 30.10971 |
| 19.32754 | 20.38663 |
| 19.34786 | 20.77996 |
| 19.36818 | 23.01395 |
| 19.3885 | 26.71155 |
| 19.40882 | 26.44826 |
| 19.42914 | 25.70783 |
| 19.44946 | 25.82672 |
| 19.46978 | 23.04005 |
| 19.49011 | 19.60322 |
| 19.51043 | 19.18979 |
| 19.53075 | 11.75068 |
| 19.55107 | 11.2281 |
| 19.57139 | 8.26042 |
| 19.59171 | 15.30123 |
| 19.61203 | 13.26889 |
| 19.63235 | 12.36098 |
| 19.65267 | 20.20064 |
| 19.673 | 19.14295 |
| 19.69332 | 22.66439 |
| 19.71364 | 25.01996 |
| 19.73396 | 26.87092 |
| 19.75428 | 26.02863 |
| 19.7746 | 27.78956 |
| 19.79492 | 31.96262 |
| 19.81524 | 35.05677 |
| 19.83557 | 40.83572 |
| 19.85589 | 43.5907 |
| 19.87621 | 49.65716 |
| 19.89653 | 50.08345 |
| 19.91685 | 58.53884 |
| 19.93717 | 56.39512 |
| 19.95749 | 61.84464 |
| 19.97781 | 58.53241 |
| 19.99814 | 55.73099 |
| 20.01846 | 49.50719 |
| 20.03878 | 49.78385 |
| 20.0591 | 60.56864 |
| 20.07942 | 59.65297 |
| 20.09974 | 59.55478 |
| 20.12006 | 59.30199 |
| 20.14038 | 52.96136 |
| 20.1607 | 48.43147 |
| 20.18103 | 21.84345 |
| 20.20135 | 19.37534 |
| 20.22167 | 20.05415 |
| 20.24199 | 16.57946 |
| 20.26231 | 14.79635 |
| 20.28263 | 8.99741 |
| 20.30295 | 1.84008 |
| 20.32327 | 2.76771 |
| 20.3436 | -1.27202 |
| 20.36392 | 0.21025 |
| 20.38424 | 0.62614 |
| 20.40456 | 2.40486 |
| 20.42488 | 3.4027 |
| 20.4452 | 3.34165 |
| 20.46552 | 7.48927 |
| 20.48584 | 5.2765 |
| 20.50617 | 1.60236 |
| 20.52649 | 1.38762 |
| 20.54681 | 4.58644 |
| 20.56713 | 12.6595 |
| 20.58745 | 11.92397 |
| 20.60777 | 17.78057 |
| 20.62809 | 20.7964 |
| 20.64841 | 22.89806 |
| 20.66874 | 24.6959 |
| 20.68906 | 25.4297 |
| 20.70938 | 31.3455 |
| 20.7297 | 27.24834 |
| 20.75002 | 28.36973 |
| 20.77034 | 24.9762 |
| 20.79066 | 33.39105 |
| 20.81098 | 34.89532 |
| 20.8313 | 35.36585 |
| 20.85163 | 32.89018 |
| 20.87195 | 40.34169 |
| 20.89227 | 34.10461 |
| 20.91259 | 29.46504 |
| 20.93291 | 35.04215 |
| 20.95323 | 32.25921 |
| 20.97355 | 34.93573 |
| 20.99387 | 32.13775 |
| 21.0142 | 31.87127 |
| 21.03452 | 33.60913 |
| 21.05484 | 40.59393 |
| 21.07516 | 40.54966 |
| 21.09548 | 33.64182 |
| 21.1158 | 33.07027 |
| 21.13612 | 26.77879 |
| 21.15644 | 21.2227 |
| 21.17677 | 12.40229 |
| 21.19709 | 1.27494 |
| 21.21741 | 18.33457 |
| 21.23773 | 19.157 |
| 21.25805 | 15.2892 |
| 21.27837 | 16.02393 |
| 21.29869 | 17.53432 |
| 21.31901 | 18.1514 |
| 21.33934 | 24.20955 |
| 21.35966 | 27.07633 |
| 21.37998 | 34.64858 |
| 21.4003 | 41.8885 |
| 21.42062 | 47.59622 |
| 21.44094 | 43.35449 |
| 21.46126 | 42.43511 |
| 21.48158 | 38.75316 |
| 21.5019 | 36.56597 |
| 21.52223 | 36.99398 |
| 21.54255 | 46.27328 |
| 21.56287 | 53.80491 |
| 21.58319 | 56.49474 |
| 21.60351 | 49.67757 |
| 21.62383 | 52.1472 |
| 21.64415 | 51.79863 |
| 21.66447 | 47.31103 |
| 21.6848 | 46.68067 |
| 21.70512 | 46.33637 |
| 21.72544 | 44.82005 |
| 21.74576 | 37.11318 |
| 21.76608 | 45.31494 |
| 21.7864 | 44.6154 |
| 21.80672 | 45.70239 |
| 21.82704 | 41.01326 |
| 21.84737 | 42.11084 |
| 21.86769 | 37.13067 |
| 21.88801 | 42.82664 |
| 21.90833 | 39.51283 |
| 21.92865 | 46.04554 |
| 21.94897 | 49.58206 |
| 21.96929 | 48.90099 |
| 21.98961 | 54.00293 |
| 22.00994 | 53.37861 |
| 22.03026 | 52.09443 |
| 22.05058 | 46.45964 |
| 22.0709 | 47.60397 |
| 22.09122 | 36.88253 |
| 22.11154 | 36.35148 |
| 22.13186 | 34.22133 |
| 22.15218 | 32.01708 |
| 22.1725 | 27.41902 |
| 22.19283 | 22.72021 |
| 22.21315 | 28.85084 |
| 22.23347 | 28.91939 |
| 22.25379 | 33.78404 |
| 22.27411 | 33.48206 |
| 22.29443 | 36.44055 |
| 22.31475 | 42.61118 |
| 22.33507 | 36.57993 |
| 22.3554 | 42.43425 |
| 22.37572 | 40.71067 |
| 22.39604 | 45.72926 |
| 22.41636 | 44.81467 |
| 22.43668 | 46.11177 |
| 22.457 | 46.44848 |
| 22.47732 | 53.94564 |
| 22.49764 | 56.76985 |
| 22.51797 | 67.41055 |
| 22.53829 | 74.58746 |
| 22.55861 | 78.27039 |
| 22.57893 | 84.20434 |
| 22.59925 | 83.60309 |
| 22.61957 | 79.51919 |
| 22.63989 | 86.42696 |
| 22.66021 | 86.6908 |
| 22.68053 | 84.31347 |
| 22.70086 | 82.20344 |
| 22.72118 | 81.2712 |
| 22.7415 | 80.47135 |
| 22.76182 | 77.22482 |
| 22.78214 | 75.14453 |
| 22.80246 | 68.78975 |
| 22.82278 | 63.22619 |
| 22.8431 | 63.59818 |
| 22.86343 | 66.73865 |
| 22.88375 | 60.40398 |
| 22.90407 | 61.55077 |
| 22.92439 | 53.30269 |
| 22.94471 | 48.10638 |
| 22.96503 | 42.70575 |
| 22.98535 | 40.57961 |
| 23.00567 | 33.49013 |
| 23.026 | 25.75939 |
| 23.04632 | 22.0443 |
| 23.06664 | 12.12347 |
| 23.08696 | 12.05654 |
| 23.10728 | 3.67836 |
| 23.1276 | 3.98676 |
| 23.14792 | 0.66654 |
| 23.16824 | 0.45074 |
| 23.18857 | -0.15435 |
| 23.20889 | 1.66238 |
| 23.22921 | 5.24262 |
| 23.24953 | -2.14875 |
| 23.26985 | 3.25452 |
| 23.29017 | 2.0116 |
| 23.31049 | 3.59716 |
| 23.33081 | 4.71093 |
| 23.35113 | 5.16213 |
| 23.37146 | 11.81561 |
| 23.39178 | 13.28667 |
| 23.4121 | 19.17218 |
| 23.43242 | 19.93572 |
| 23.45274 | 24.5119 |
| 23.47306 | 31.93953 |
| 23.49338 | 41.40623 |
| 23.5137 | 43.06113 |
| 23.53403 | 48.60968 |
| 23.55435 | 48.38857 |
| 23.57467 | 52.06016 |
| 23.59499 | 57.8551 |
| 23.61531 | 52.91814 |
| 23.63563 | 51.68414 |
| 23.65595 | 51.78167 |
| 23.67627 | 52.69053 |
| 23.6966 | 54.31457 |
| 23.71692 | 52.82457 |
| 23.73724 | 56.15398 |
| 23.75756 | 54.74142 |
| 23.77788 | 49.10567 |
| 23.7982 | 48.71522 |
| 23.81852 | 57.53153 |
| 23.83884 | 57.32278 |
| 23.85917 | 68.38263 |
| 23.87949 | 65.23425 |
| 23.89981 | 59.70679 |
| 23.92013 | 61.23134 |
| 23.94045 | 56.87068 |
| 23.96077 | 55.89736 |
| 23.98109 | 57.152 |
| 24.00141 | 60.34567 |
| 24.02173 | 61.12728 |
| 24.04206 | 64.34461 |
| 24.06238 | 64.78132 |
| 24.0827 | 60.79195 |
| 24.10302 | 69.64243 |
| 24.12334 | 69.1242 |
| 24.14366 | 74.50543 |
| 24.16398 | 78.83384 |
| 24.1843 | 83.35962 |
| 24.20463 | 90.22967 |
| 24.22495 | 84.02586 |
| 24.24527 | 82.91356 |
| 24.26559 | 79.99511 |
| 24.28591 | 80.15632 |
| 24.30623 | 81.8455 |
| 24.32655 | 83.21106 |
| 24.34687 | 85.45877 |
| 24.3672 | 80.13623 |
| 24.38752 | 76.10057 |
| 24.40784 | 79.46233 |
| 24.42816 | 82.21894 |
| 24.44848 | 83.08371 |
| 24.4688 | 81.58243 |
| 24.48912 | 85.0653 |
| 24.50944 | 77.42108 |
| 24.52977 | 70.53686 |
| 24.55009 | 66.11108 |
| 24.57041 | 62.35717 |
| 24.59073 | 55.66607 |
| 24.61105 | 50.97479 |
| 24.63137 | 42.20289 |
| 24.65169 | 36.55446 |
| 24.67201 | 37.4127 |
| 24.69233 | 33.86307 |
| 24.71266 | 26.12831 |
| 24.73298 | 21.47142 |
| 24.7533 | 21.20239 |
| 24.77362 | 19.40361 |
| 24.79394 | 18.52642 |
| 24.81426 | 20.50347 |
| 24.83458 | 16.60487 |
| 24.8549 | 11.53364 |
| 24.87523 | 17.04055 |
| 24.89555 | 13.0378 |
| 24.91587 | 13.14483 |
| 24.93619 | 12.69887 |
| 24.95651 | 10.55209 |
| 24.97683 | 11.72396 |
| 24.99715 | 20.19109 |
| 25.01747 | 18.35297 |
| 25.0378 | 20.49244 |
| 25.05812 | 23.69018 |
| 25.07844 | 23.8173 |
| 25.09876 | 27.39613 |
| 25.11908 | 30.5311 |
| 25.1394 | 29.68155 |
| 25.15972 | 33.72276 |
| 25.18004 | 32.17281 |
| 25.20037 | 29.74199 |
| 25.22069 | 28.00574 |
| 25.24101 | 26.97161 |
| 25.26133 | 33.80115 |
| 25.28165 | 35.38768 |
| 25.30197 | 40.20013 |
| 25.32229 | 42.696 |
| 25.34261 | 43.426 |
| 25.36293 | 42.72932 |
| 25.38326 | 45.1851 |
| 25.40358 | 50.80292 |
| 25.4239 | 55.98123 |
| 25.44422 | 57.09036 |
| 25.46454 | 53.17977 |
| 25.48486 | 53.52381 |
| 25.50518 | 53.05475 |
| 25.5255 | 48.22062 |
| 25.54583 | 48.4557 |
| 25.56615 | 40.21957 |
| 25.58647 | 39.32854 |
| 25.60679 | 29.38438 |
| 25.62711 | 29.10243 |
| 25.64743 | 27.1763 |
| 25.66775 | 29.46828 |
| 25.68807 | 28.1989 |
| 25.7084 | 20.21371 |
| 25.72872 | 21.57002 |
| 25.74904 | 24.90264 |
| 25.76936 | 26.47363 |
| 25.78968 | 26.34085 |
| 25.81 | 19.98719 |
| 25.83032 | 20.27192 |
| 25.85064 | 22.53774 |
| 25.87096 | 24.83428 |
| 25.89129 | 31.24623 |
| 25.91161 | 29.88076 |
| 25.93193 | 24.45992 |
| 25.95225 | 27.84501 |
| 25.97257 | 28.84562 |
| 25.99289 | 30.66463 |
| 26.01321 | 38.19851 |
| 26.03353 | 36.80716 |
| 26.05386 | 43.54111 |
| 26.07418 | 44.94398 |
| 26.0945 | 47.34828 |
| 26.11482 | 54.49802 |
| 26.13514 | 58.2597 |
| 26.15546 | 63.97623 |
| 26.17578 | 69.71403 |
| 26.1961 | 75.8707 |
| 26.21643 | 81.19467 |
| 26.23675 | 88.90989 |
| 26.25707 | 89.74013 |
| 26.27739 | 89.10329 |
| 26.29771 | 87.20467 |
| 26.31803 | 97.25395 |
| 26.33835 | 94.97026 |
| 26.35867 | 101.31997 |
| 26.379 | 105.46103 |
| 26.39932 | 108.63433 |
| 26.41964 | 105.78707 |
| 26.43996 | 108.03104 |
| 26.46028 | 110.63304 |
| 26.4806 | 114.01855 |
| 26.50092 | 115.83448 |
| 26.52124 | 110.9311 |
| 26.54156 | 114.15392 |
| 26.56189 | 109.73279 |
| 26.58221 | 107.52579 |
| 26.60253 | 111.83277 |
| 26.62285 | 107.54385 |
| 26.64317 | 116.10741 |
| 26.66349 | 114.80638 |
| 26.68381 | 118.07402 |
| 26.70413 | 112.00501 |
| 26.72446 | 117.23796 |
| 26.74478 | 116.97872 |
| 26.7651 | 115.10808 |
| 26.78542 | 110.54278 |
| 26.80574 | 104.74362 |
| 26.82606 | 100.50105 |
| 26.84638 | 100.46209 |
| 26.8667 | 97.34218 |
| 26.88703 | 89.41727 |
| 26.90735 | 87.60569 |
| 26.92767 | 75.77649 |
| 26.94799 | 69.32603 |
| 26.96831 | 71.78609 |
| 26.98863 | 66.11128 |
| 27.00895 | 63.29291 |
| 27.02927 | 65.79748 |
| 27.0496 | 57.56039 |
| 27.06992 | 51.90166 |
| 27.09024 | 53.03683 |
| 27.11056 | 51.96329 |
| 27.13088 | 50.10315 |
| 27.1512 | 48.6973 |
| 27.17152 | 44.35913 |
| 27.19184 | 40.65211 |
| 27.21216 | 37.76729 |
| 27.23249 | 28.41559 |
| 27.25281 | 21.91215 |
| 27.27313 | 15.97095 |
| 27.29345 | 11.74748 |
| 27.31377 | 7.16275 |
| 27.33409 | 9.97988 |
| 27.35441 | 4.04527 |
| 27.37473 | 4.29254 |
| 27.39506 | -0.88484 |
| 27.41538 | -0.96715 |
| 27.4357 | -1.03074 |
| 27.45602 | 1.77573 |
| 27.47634 | 3.89463 |
| 27.49666 | 5.33537 |
| 27.51698 | -0.22399 |
| 27.5373 | 5.55888 |
| 27.55763 | 14.73779 |
| 27.57795 | 10.82503 |
| 27.59827 | 14.96525 |
| 27.61859 | 17.95764 |
| 27.63891 | 20.10876 |
| 27.65923 | 22.19223 |
| 27.67955 | 19.02729 |
| 27.69987 | 20.47449 |
| 27.7202 | 15.46755 |
| 27.74052 | 22.25046 |
| 27.76084 | 22.95339 |
| 27.78116 | 27.55717 |
| 27.80148 | 27.79581 |
| 27.8218 | 24.72876 |
| 27.84212 | 26.87138 |
| 27.86244 | 20.01665 |
| 27.88276 | 21.39473 |
| 27.90309 | 20.38758 |
| 27.92341 | 19.26262 |
| 27.94373 | 15.79656 |
| 27.96405 | 21.01377 |
| 27.98437 | 24.19721 |
| 28.00469 | 21.29119 |
| 28.02501 | 20.03662 |
| 28.04533 | 21.86353 |
| 28.06566 | 15.72987 |
| 28.08598 | 15.40071 |
| 28.1063 | 19.10963 |
| 28.12662 | 19.85 |
| 28.14694 | 23.65308 |
| 28.16726 | 28.26434 |
| 28.18758 | 23.86778 |
| 28.2079 | 22.1981 |
| 28.22823 | 16.77551 |
| 28.24855 | 8.18743 |
| 28.26887 | 3.08418 |
| 28.28919 | 5.38303 |
| 28.30951 | 6.28389 |
| 28.32983 | 10.97293 |
| 28.35015 | 12.22765 |
| 28.37047 | 13.9214 |
| 28.39079 | 15.94487 |
| 28.41112 | 20.77342 |
| 28.43144 | 27.3267 |
| 28.45176 | 27.36953 |
| 28.47208 | 32.0787 |
| 28.4924 | 34.70828 |
| 28.51272 | 40.60821 |
| 28.53304 | 44.52659 |
| 28.55336 | 49.32588 |
| 28.57369 | 49.4586 |
| 28.59401 | 45.60776 |
| 28.61433 | 48.31797 |
| 28.63465 | 50.90107 |
| 28.65497 | 51.34805 |
| 28.67529 | 51.41261 |
| 28.69561 | 53.18664 |
| 28.71593 | 55.70984 |
| 28.73626 | 58.71006 |
| 28.75658 | 63.72196 |
| 28.7769 | 64.31673 |
| 28.79722 | 56.90503 |
| 28.81754 | 46.88119 |
| 28.83786 | 43.71845 |
| 28.85818 | 45.09682 |
| 28.8785 | 44.07694 |
| 28.89883 | 46.41531 |
| 28.91915 | 43.87448 |
| 28.93947 | 40.54931 |
| 28.95979 | 34.5248 |
| 28.98011 | 37.90188 |
| 29.00043 | 34.49817 |
| 29.02075 | 28.17864 |
| 29.04107 | 29.35177 |
| 29.06139 | 30.37741 |
| 29.08172 | 32.20877 |
| 29.10204 | 30.76002 |
| 29.12236 | 30.29229 |
| 29.14268 | 28.64638 |
| 29.163 | 24.74084 |
| 29.18332 | 19.1461 |
| 29.20364 | 12.98097 |
| 29.22396 | 8.3389 |
| 29.24429 | 5.62727 |
| 29.26461 | -0.06992 |
| 29.28493 | 2.58245 |
| 29.30525 | 5.33876 |
| 29.32557 | 8.71216 |
| 29.34589 | 4.11658 |
| 29.36621 | 2.33244 |
| 29.38653 | 5.31366 |
| 29.40686 | 4.71859 |
| 29.42718 | 3.01149 |
| 29.4475 | -0.81753 |
| 29.46782 | -0.73518 |
| 29.48814 | -1.43841 |
| 29.50846 | -4.35886 |
| 29.52878 | -0.59997 |
| 29.5491 | -2.9506 |
| 29.56943 | -1.10217 |
| 29.58975 | 2.01761 |
| 29.61007 | 0.95481 |
| 29.63039 | 3.42114 |
| 29.65071 | 6.84835 |
| 29.67103 | 6.55465 |
| 29.69135 | 6.59325 |
| 29.71167 | 7.98691 |
| 29.73199 | 8.06522 |
| 29.75232 | 7.54088 |
| 29.77264 | 11.85667 |
| 29.79296 | 13.32736 |
| 29.81328 | 11.0092 |
| 29.8336 | 17.46678 |
| 29.85392 | 30.09516 |
| 29.87424 | 41.17217 |
| 29.89456 | 45.69757 |
| 29.91489 | 44.95756 |
| 29.93521 | 47.91166 |
| 29.95553 | 48.61307 |
| 29.97585 | 46.3912 |
| 29.99617 | 52.75875 |
| 30.01649 | 49.24926 |
| 30.03681 | 51.12864 |
| 30.05713 | 55.9532 |
| 30.07746 | 53.19643 |
| 30.09778 | 50.39448 |
| 30.1181 | 49.74886 |
| 30.13842 | 48.7244 |
| 30.15874 | 49.73257 |
| 30.17906 | 47.56179 |
| 30.19938 | 42.8935 |
| 30.2197 | 40.64001 |
| 30.24003 | 36.81877 |
| 30.26035 | 37.54736 |
| 30.28067 | 34.61525 |
| 30.30099 | 35.07163 |
| 30.32131 | 33.29178 |
| 30.34163 | 32.01765 |
| 30.36195 | 22.45435 |
| 30.38227 | 16.5182 |
| 30.40259 | 15.52599 |
| 30.42292 | 11.19993 |
| 30.44324 | 8.8082 |
| 30.46356 | 6.13384 |
| 30.48388 | 2.98488 |
| 30.5042 | -2.80465 |
| 30.52452 | -1.96221 |
| 30.54484 | -3.63693 |
| 30.56516 | -4.19692 |
| 30.58549 | -7.33573 |
| 30.60581 | -6.19827 |
| 30.62613 | -6.05435 |
| 30.64645 | -7.36457 |
| 30.66677 | -4.53437 |
| 30.68709 | -5.75229 |
| 30.70741 | -2.41577 |
| 30.72773 | -2.51539 |
| 30.74806 | -7.5179 |
| 30.76838 | -2.80339 |
| 30.7887 | 1.19312 |
| 30.80902 | 1.28682 |
| 30.82934 | 5.01639 |
| 30.84966 | 9.54967 |
| 30.86998 | 14.42335 |
| 30.8903 | 8.36343 |
| 30.91062 | 0.00892 |
| 30.93095 | -2.75836 |
| 30.95127 | -3.66112 |
| 30.97159 | -4.62651 |
| 30.99191 | -4.44753 |
| 31.01223 | -3.24222 |
| 31.03255 | -5.97429 |
| 31.05287 | -1.7203 |
| 31.07319 | 1.48147 |
| 31.09352 | 3.08463 |
| 31.11384 | 5.23778 |
| 31.13416 | 10.58918 |
| 31.15448 | 6.69841 |
| 31.1748 | 5.01688 |
| 31.19512 | 5.02404 |
| 31.21544 | 8.03537 |
| 31.23576 | 9.70829 |
| 31.25609 | 14.66571 |
| 31.27641 | 18.19153 |
| 31.29673 | 20.93667 |
| 31.31705 | 22.81343 |
| 31.33737 | 33.05755 |
| 31.35769 | 41.12332 |
| 31.37801 | 48.77065 |
| 31.39833 | 50.47661 |
| 31.41866 | 59.12706 |
| 31.43898 | 60.6315 |
| 31.4593 | 68.45382 |
| 31.47962 | 73.36479 |
| 31.49994 | 76.25589 |
| 31.52026 | 84.08103 |
| 31.54058 | 90.13106 |
| 31.5609 | 92.81768 |
| 31.58122 | 98.2753 |
| 31.60155 | 101.70717 |
| 31.62187 | 103.76422 |
| 31.64219 | 106.79686 |
| 31.66251 | 116.90144 |
| 31.68283 | 128.31765 |
| 31.70315 | 142.48172 |
| 31.72347 | 157.24951 |
| 31.74379 | 173.13673 |
| 31.76412 | 188.54397 |
| 31.78444 | 210.92957 |
| 31.80476 | 230.63188 |
| 31.82508 | 245.32194 |
| 31.8454 | 256.36325 |
| 31.86572 | 268.38126 |
| 31.88604 | 279.15729 |
| 31.90636 | 283.27464 |
| 31.92669 | 293.06945 |
| 31.94701 | 299.38994 |
| 31.96733 | 301.19753 |
| 31.98765 | 305.52247 |
| 32.00797 | 512.1093 |
| 32.02829 | 304.74285 |
| 32.04861 | 301.25862 |
| 32.06893 | 289.54387 |
| 32.08926 | 287.49839 |
| 32.10958 | 284.34115 |
| 32.1299 | 280.78038 |
| 32.15022 | 275.93969 |
| 32.17054 | 267.02713 |
| 32.19086 | 261.23806 |
| 32.21118 | 254.54923 |
| 32.2315 | 242.11589 |
| 32.25182 | 231.54608 |
| 32.27215 | 225.60178 |
| 32.29247 | 213.25799 |
| 32.31279 | 204.05192 |
| 32.35343 | 193.33292 |
| 32.37375 | 188.62935 |
| 32.39407 | 168.70504 |
| 32.41439 | 152.01766 |
| 32.43472 | 137.57237 |
| 32.45504 | 124.84227 |
| 32.47536 | 108.92097 |
| 32.49568 | 96.08717 |
| 32.516 | 77.91504 |
| 32.53632 | 61.44184 |
| 32.55664 | 50.84837 |
| 32.57696 | 34.38415 |
| 32.59729 | 20.67696 |
| 32.61761 | 9.77931 |
| 32.63793 | 4.53426 |
| 32.65825 | 0.26623 |
| 32.67857 | 6.94935 |
| 32.69889 | 15.18296 |
| 32.71921 | 19.65914 |
| 32.73953 | 26.66264 |
| 32.75986 | 27.28282 |
| 32.78018 | 31.74564 |
| 32.8005 | 32.04966 |
| 32.82082 | 35.79676 |
| 32.84114 | 33.86657 |
| 32.86146 | 25.21795 |
| 32.88178 | 22.94984 |
| 32.9021 | 27.94619 |
| 32.92242 | 24.94605 |
| 32.94275 | 19.44344 |
| 32.96307 | 18.38404 |
| 32.98339 | 15.45706 |
| 33.00371 | 15.5352 |
| 33.02403 | 15.97753 |
| 33.04435 | 9.44377 |
| 33.06467 | 10.75568 |
| 33.08499 | 9.99061 |
| 33.10532 | 9.95484 |
| 33.12564 | 24.74372 |
| 33.14596 | 26.57462 |
| 33.16628 | 27.49209 |
| 33.1866 | 24.27058 |
| 33.20692 | 24.31717 |
| 33.22724 | 28.75679 |
| 33.24756 | 26.57278 |
| 33.26789 | 24.40526 |
| 33.28821 | 25.82634 |
| 33.30853 | 27.05598 |
| 33.32885 | 28.19803 |
| 33.34917 | 33.26606 |
| 33.36949 | 34.15604 |
| 33.38981 | 36.09107 |
| 33.41013 | 34.73754 |
| 33.43046 | 39.50346 |
| 33.45078 | 41.81594 |
| 33.4711 | 39.83406 |
| 33.49142 | 42.71012 |
| 33.51174 | 42.88391 |
| 33.53206 | 45.18829 |
| 33.55238 | 46.83077 |
| 33.5727 | 45.21005 |
| 33.59302 | 44.18364 |
| 33.61335 | 44.05992 |
| 33.63367 | 45.84945 |
| 33.65399 | 47.36814 |
| 33.67431 | 48.93798 |
| 33.69463 | 53.25132 |
| 33.71495 | 58.20382 |
| 33.73527 | 53.51108 |
| 33.75559 | 52.52937 |
| 33.77592 | 51.37128 |
| 33.79624 | 55.27555 |
| 33.81656 | 52.03996 |
| 33.83688 | 50.78376 |
| 33.8572 | 49.87509 |
| 33.87752 | 51.93563 |
| 33.89784 | 55.08369 |
| 33.91816 | 56.9823 |
| 33.93849 | 58.55027 |
| 33.95881 | 57.23296 |
| 33.97913 | 58.38758 |
| 33.99945 | 56.95952 |
| 34.01977 | 55.42417 |
| 34.04009 | 50.82686 |
| 34.06041 | 49.78916 |
| 34.08073 | 53.15965 |
| 34.10105 | 51.73531 |
| 34.12138 | 50.06471 |
| 34.1417 | 50.14597 |
| 34.16202 | 51.73253 |
| 34.18234 | 48.82438 |
| 34.20266 | 45.11366 |
| 34.22298 | 48.78936 |
| 34.2433 | 47.13849 |
| 34.26362 | 41.55691 |
| 34.28395 | 42.46874 |
| 34.30427 | 44.72245 |
| 34.32459 | 43.56199 |
| 34.34491 | 46.52445 |
| 34.38555 | 43.97212 |
| 34.40587 | 43.20738 |
| 34.42619 | 43.79856 |
| 34.44652 | 39.62401 |
| 34.46684 | 37.75852 |
| 34.48716 | 35.4275 |
| 34.50748 | 34.32013 |
| 34.5278 | 32.80023 |
| 34.54812 | 25.90678 |
| 34.56844 | 24.90559 |
| 34.58876 | 20.6242 |
| 34.60909 | 22.77189 |
| 34.62941 | 26.27687 |
| 34.64973 | 24.69129 |
| 34.67005 | 25.43675 |
| 34.69037 | 23.69263 |
| 34.71069 | 20.10571 |
| 34.73101 | 18.55661 |
| 34.75133 | 18.11903 |
| 34.77165 | 15.53389 |
| 34.79198 | 16.53349 |
| 34.8123 | 16.58307 |
| 34.83262 | 13.91576 |
| 34.85294 | 10.64227 |
| 34.87326 | 11.19316 |
| 34.89358 | 12.96259 |
| 34.9139 | 12.5053 |
| 34.93422 | 13.65025 |
| 34.95455 | 15.36288 |
| 34.97487 | 17.85191 |
| 34.99519 | 14.07359 |
| 35.01551 | 15.2892 |
| 35.03583 | 11.82934 |
| 35.05615 | 12.0712 |
| 35.07647 | 13.84565 |
| 35.09679 | 18.80199 |
| 35.11712 | 24.81572 |
| 35.13744 | 28.73058 |
| 35.15776 | 31.91683 |
| 35.17808 | 34.79248 |
| 35.1984 | 39.93958 |
| 35.21872 | 39.93271 |
| 35.23904 | 37.88032 |
| 35.25936 | 42.36278 |
| 35.27969 | 41.01527 |
| 35.30001 | 45.69365 |
| 35.32033 | 48.18902 |
| 35.34065 | 44.55032 |
| 35.36097 | 41.82745 |
| 35.38129 | 44.18457 |
| 35.40161 | 43.51926 |
| 35.42193 | 44.79278 |
| 35.44225 | 43.41033 |
| 35.46258 | 43.33001 |
| 35.4829 | 44.05519 |
| 35.50322 | 43.8018 |
| 35.52354 | 43.88233 |
| 35.54386 | 39.32464 |
| 35.56418 | 38.98114 |
| 35.5845 | 36.95215 |
| 35.60482 | 40.59468 |
| 35.62515 | 38.2395 |
| 35.64547 | 30.3864 |
| 35.66579 | 21.16355 |
| 35.68611 | 17.43396 |
| 35.70643 | 12.6753 |
| 35.72675 | 6.06885 |
| 35.74707 | 7.88631 |
| 35.76739 | 5.40858 |
| 35.78772 | 5.18181 |
| 35.80804 | 2.51539 |
| 35.82836 | -0.06141 |
| 35.84868 | -0.67706 |
| 35.869 | -0.05018 |
| 35.88932 | -1.66951 |
| 35.90964 | -3.10384 |
| 35.92996 | -5.94224 |
| 35.95029 | -6.32426 |
| 35.97061 | -3.16458 |
| 35.99093 | -2.26347 |
| 36.01125 | -4.55037 |
| 36.03157 | -6.28875 |
| 36.05189 | -5.74232 |
| 36.07221 | 1.97748 |
| 36.09253 | 4.47818 |
| 36.11285 | 9.55214 |
| 36.13318 | 9.59907 |
| 36.1535 | 6.37043 |
| 36.17382 | 3.38889 |
| 36.19414 | 0.86972 |
| 36.21446 | -1.11815 |
| 36.23478 | -2.13114 |
| 36.2551 | -7.12421 |
| 36.27542 | -4.72218 |
| 36.29575 | -2.57655 |
| 36.31607 | -2.88028 |
| 36.33639 | -3.18273 |
| 36.35671 | -9.187 |
| 36.37703 | -8.21097 |
| 36.41767 | -5.92992 |
| 36.43799 | -5.8121 |
| 36.45832 | -6.08937 |
| 36.47864 | -8.03213 |
| 36.49896 | -4.56653 |
| 36.51928 | -1.44052 |
| 36.5396 | -0.25679 |
| 36.55992 | 2.68517 |
| 36.58024 | 5.82254 |
| 36.60056 | 8.70737 |
| 36.62088 | 8.95758 |
| 36.64121 | 14.76664 |
| 36.66153 | 13.417 |
| 36.68185 | 16.10786 |
| 36.70217 | 16.60021 |
| 36.72249 | 19.27654 |
| 36.74281 | 20.07864 |
| 36.76313 | 20.71964 |
| 36.78345 | 24.44975 |
| 36.80378 | 27.76817 |
| 36.8241 | 31.80049 |
| 36.84442 | 27.86935 |
| 36.86474 | 29.15316 |
| 36.88506 | 36.5861 |
| 36.90538 | 38.40512 |
| 36.9257 | 40.15359 |
| 36.94602 | 40.84853 |
| 36.96635 | 43.66904 |
| 36.98667 | 49.42562 |
| 37.00699 | 47.77679 |
| 37.02731 | 44.90214 |
| 37.04763 | 45.68636 |
| 37.06795 | 50.48445 |
| 37.08827 | 56.96846 |
| 37.10859 | 57.27238 |
| 37.12892 | 51.25676 |
| 37.14924 | 52.65539 |
| 37.16956 | 47.47665 |
| 37.18988 | 43.73007 |
| 37.2102 | 42.00849 |
| 37.23052 | 38.58304 |
| 37.25084 | 35.9449 |
| 37.27116 | 34.70729 |
| 37.29148 | 25.45853 |
| 37.31181 | 20.59897 |
| 37.33213 | 15.4962 |
| 37.35245 | 4.90034 |
| 37.37277 | 2.67861 |
| 37.39309 | -5.90879 |
| 37.41341 | -5.19165 |
| 37.43373 | -4.40839 |
| 37.45405 | -3.30796 |
| 37.47438 | -0.11176 |
| 37.4947 | -0.21736 |
| 37.51502 | 5.09785 |
| 37.53534 | 2.93035 |
| 37.55566 | -0.04714 |
| 37.57598 | -2.05648 |
| 37.5963 | -3.10892 |
| 37.61662 | -0.74317 |
| 37.63695 | -0.87684 |
| 37.65727 | -3.10101 |
| 37.67759 | -6.57233 |
| 37.69791 | -10.25014 |
| 37.71823 | -9.6686 |
| 37.73855 | -4.49669 |
| 37.75887 | -0.7825 |
| 37.77919 | 0.38026 |
| 37.79952 | 0.23721 |
| 37.81984 | 2.40469 |
| 37.84016 | 0.54215 |
| 37.86048 | 2.40945 |
| 37.8808 | 8.60998 |
| 37.90112 | 13.67136 |
| 37.92144 | 15.54575 |
| 37.94176 | 19.41965 |
| 37.96208 | 26.20272 |
| 37.98241 | 32.5474 |
| 38.00273 | 34.59584 |
| 38.02305 | 36.88699 |
| 38.04337 | 49.18345 |
| 38.06369 | 52.64379 |
| 38.08401 | 57.96269 |
| 38.10433 | 54.85679 |
| 38.12465 | 51.00779 |
| 38.14498 | 53.1805 |
| 38.1653 | 59.7717 |
| 38.18562 | 57.53284 |
| 38.20594 | 62.02869 |
| 38.22626 | 61.28979 |
| 38.24658 | 60.29698 |
| 38.2669 | 58.92749 |
| 38.28722 | 53.50489 |
| 38.30755 | 52.18157 |
| 38.32787 | 55.41188 |
| 38.34819 | 53.18926 |
| 38.36851 | 55.29216 |
| 38.38883 | 55.13922 |
| 38.40915 | 54.37555 |
| 38.42947 | 56.53939 |
| 38.44979 | 56.47205 |
| 38.47012 | 53.54747 |
| 38.49044 | 53.35896 |
| 38.51076 | 48.11654 |
| 38.53108 | 46.71529 |
| 38.5514 | 36.97486 |
| 38.57172 | 40.5646 |
| 38.59204 | 33.51022 |
| 38.61236 | 36.86497 |
| 38.63268 | 34.75667 |
| 38.65301 | 25.1651 |
| 38.67333 | 19.99926 |
| 38.69365 | 19.84238 |
| 38.71397 | 21.74852 |
| 38.73429 | 20.35492 |
| 38.75461 | 17.86797 |
| 38.77493 | 11.26356 |
| 38.79525 | 6.4483 |
| 38.81558 | 3.85455 |
| 38.8359 | 3.87282 |
| 38.85622 | 3.80835 |
| 38.87654 | 5.57035 |
| 38.89686 | 9.24782 |
| 38.91718 | 8.50109 |
| 38.9375 | 12.33805 |
| 38.95782 | 14.62923 |
| 38.97815 | 11.6386 |
| 38.99847 | 14.51374 |
| 39.01879 | 10.7014 |
| 39.03911 | 17.28615 |
| 39.05943 | 18.67844 |
| 39.07975 | 15.15837 |
| 39.10007 | 18.80224 |
| 39.12039 | 16.76641 |
| 39.14071 | 20.63886 |
| 39.16104 | 20.62878 |
| 39.18136 | 20.32803 |
| 39.20168 | 23.34295 |
| 39.222 | 27.23604 |
| 39.24232 | 24.30919 |
| 39.26264 | 27.1556 |
| 39.28296 | 31.62401 |
| 39.30328 | 31.35271 |
| 39.32361 | 39.24406 |
| 39.34393 | 38.7248 |
| 39.36425 | 38.79141 |
| 39.38457 | 42.91345 |
| 39.40489 | 41.45482 |
| 39.42521 | 41.20428 |
| 39.44553 | 40.51935 |
| 39.46585 | 40.636 |
| 39.48618 | 40.36432 |
| 39.5065 | 41.01207 |
| 39.52682 | 32.76514 |
| 39.54714 | 33.29474 |
| 39.56746 | 32.75754 |
| 39.58778 | 38.94548 |
| 39.6081 | 34.82979 |
| 39.62842 | 34.78171 |
| 39.64875 | 31.08482 |
| 39.66907 | 30.21957 |
| 39.68939 | 35.82403 |
| 39.70971 | 41.90251 |
| 39.73003 | 43.65097 |
| 39.75035 | 45.50388 |
| 39.77067 | 55.70731 |
| 39.79099 | 58.14553 |
| 39.81131 | 66.55536 |
| 39.83164 | 75.42312 |
| 39.85196 | 78.46749 |
| 39.87228 | 83.30738 |
| 39.8926 | 85.56118 |
| 39.91292 | 88.35708 |
| 39.93324 | 87.89492 |
| 39.95356 | 92.45068 |
| 39.97388 | 92.01093 |
| 39.99421 | 91.58793 |
| 40.01453 | 97.78152 |
| 40.03485 | 94.22704 |
| 40.05517 | 97.69559 |
| 40.07549 | 93.56975 |
| 40.09581 | 92.52444 |
| 40.11613 | 89.62108 |
| 40.13645 | 86.5337 |
| 40.15678 | 79.79508 |
| 40.1771 | 73.96699 |
| 40.19742 | 78.23482 |
| 40.21774 | 79.17578 |
| 40.23806 | 75.48465 |
| 40.25838 | 71.78277 |
| 40.2787 | 70.93171 |
| 40.29902 | 71.95924 |
| 40.31935 | 66.30088 |
| 40.33967 | 70.4568 |
| 40.35999 | 63.50325 |
| 40.38031 | 66.16247 |
| 40.40063 | 61.32617 |
| 40.42095 | 54.46871 |
| 40.44127 | 50.23154 |
| 40.46159 | 50.02687 |
| 40.48191 | 45.70199 |
| 40.50224 | 42.07641 |
| 40.52256 | 37.06371 |
| 40.54288 | 36.36044 |
| 40.5632 | 36.23618 |
| 40.58352 | 32.28986 |
| 40.60384 | 32.626 |
| 40.62416 | 30.54766 |
| 40.64448 | 27.74471 |
| 40.66481 | 36.26288 |
| 40.68513 | 41.69451 |
| 40.70545 | 44.95778 |
| 40.72577 | 47.00825 |
| 40.74609 | 47.34049 |
| 40.76641 | 53.99968 |
| 40.78673 | 56.97942 |
| 40.80705 | 52.89018 |
| 40.82738 | 54.90456 |
| 40.8477 | 50.81811 |
| 40.86802 | 47.70727 |
| 40.88834 | 44.19592 |
| 40.90866 | 39.44071 |
| 40.92898 | 35.25292 |
| 40.9493 | 30.99328 |
| 40.96962 | 27.20125 |
| 40.98995 | 27.9722 |
| 41.01027 | 26.06512 |
| 41.03059 | 25.63381 |
| 41.05091 | 23.21289 |
| 41.07123 | 20.77582 |
| 41.09155 | 16.5114 |
| 41.11187 | 16.39973 |
| 41.13219 | 11.63466 |
| 41.15251 | 12.73639 |
| 41.17284 | 5.2855 |
| 41.19316 | 13.57192 |
| 41.21348 | 17.10291 |
| 41.2338 | 14.62516 |
| 41.25412 | 14.07716 |
| 41.27444 | 12.32941 |
| 41.29476 | 14.04379 |
| 41.31508 | 18.0929 |
| 41.33541 | 17.99293 |
| 41.35573 | 21.57366 |
| 41.37605 | 19.98702 |
| 41.39637 | 23.02318 |
| 41.41669 | 19.28588 |
| 41.43701 | 19.30371 |
| 41.45733 | 19.26587 |
| 41.47765 | 18.47554 |
| 41.49798 | 17.12132 |
| 41.5183 | 16.67316 |
| 41.53862 | 12.01919 |
| 41.55894 | 10.1808 |
| 41.57926 | 8.91097 |
| 41.59958 | 9.43727 |
| 41.6199 | 17.54935 |
| 41.64022 | 15.7174 |
| 41.66055 | 17.94459 |
| 41.68087 | 20.49305 |
| 41.70119 | 14.04615 |
| 41.72151 | 12.64134 |
| 41.74183 | 14.73743 |
| 41.76215 | 14.14582 |
| 41.78247 | 14.18796 |
| 41.80279 | 10.23517 |
| 41.82311 | 8.6733 |
| 41.84344 | 10.88287 |
| 41.86376 | 7.49626 |
| 41.88408 | 4.71155 |
| 41.9044 | 2.01544 |
| 41.92472 | 2.55316 |
| 41.94504 | 1.89854 |
| 41.96536 | 3.91642 |
| 41.98568 | 4.6624 |
| 42.00601 | 7.43776 |
| 42.02633 | 5.67813 |
| 42.04665 | 7.18365 |
| 42.06697 | 5.44134 |
| 42.08729 | 8.75262 |
| 42.10761 | 10.12453 |
| 42.12793 | 15.94362 |
| 42.14825 | 20.69308 |
| 42.16858 | 21.54722 |
| 42.1889 | 21.84697 |
| 42.20922 | 28.94672 |
| 42.22954 | 27.76683 |
| 42.24986 | 27.46783 |
| 42.27018 | 29.78564 |
| 42.2905 | 34.60245 |
| 42.31082 | 41.28808 |
| 42.33114 | 44.50423 |
| 42.35147 | 43.87804 |
| 42.37179 | 42.89902 |
| 42.39211 | 43.61998 |
| 42.41243 | 43.27003 |
| 42.43275 | 42.51304 |
| 42.45307 | 44.82742 |
| 42.47339 | 42.23291 |
| 42.51404 | 44.22743 |
| 42.53436 | 42.4081 |
| 42.55468 | 43.39839 |
| 42.575 | 42.23283 |
| 42.59532 | 42.74384 |
| 42.61564 | 44.12189 |
| 42.63596 | 44.31139 |
| 42.65628 | 42.91322 |
| 42.67661 | 35.03459 |
| 42.69693 | 36.58661 |
| 42.71725 | 34.34358 |
| 42.73757 | 31.79749 |
| 42.75789 | 31.74614 |
| 42.77821 | 26.02984 |
| 42.79853 | 22.29517 |
| 42.81885 | 21.52515 |
| 42.83918 | 19.06776 |
| 42.8595 | 16.73276 |
| 42.87982 | 12.70672 |
| 42.90014 | 7.65305 |
| 42.92046 | 4.25558 |
| 42.94078 | 4.28646 |
| 42.9611 | 0.5249 |
| 42.98142 | -2.41159 |
| 43.00174 | -5.8968 |
| 43.02207 | -12.79175 |
| 43.04239 | -15.32256 |
| 43.06271 | -18.3528 |
| 43.08303 | -18.7191 |
| 43.10335 | -16.2172 |
| 43.12367 | -14.63089 |
| 43.14399 | -15.76456 |
| 43.16431 | -14.59478 |
| 43.18464 | -13.3214 |
| 43.20496 | -15.16379 |
| 43.22528 | -12.70073 |
| 43.2456 | -11.82751 |
| 43.26592 | -9.49179 |
| 43.28624 | -7.68025 |
| 43.30656 | -3.2205 |
| 43.32688 | 1.17526 |
| 43.34721 | 1.17417 |
| 43.36753 | 0.26146 |
| 43.38785 | -0.86143 |
| 43.40817 | -1.08872 |
| 43.42849 | 3.29585 |
| 43.44881 | 1.73028 |
| 43.46913 | 1.4933 |
| 43.48945 | 2.75138 |
| 43.50978 | 0.60946 |
| 43.5301 | 4.05096 |
| 43.55042 | 3.3796 |
| 43.57074 | 2.29804 |
| 43.59106 | 0.02435 |
| 43.61138 | -1.54632 |
| 43.6317 | 1.72154 |
| 43.65202 | 3.4991 |
| 43.67234 | 1.69524 |
| 43.69267 | 7.0318 |
| 43.71299 | 8.13478 |
| 43.73331 | 7.66331 |
| 43.75363 | 5.57684 |
| 43.77395 | 7.42675 |
| 43.79427 | 8.34042 |
| 43.81459 | 10.83568 |
| 43.83491 | 9.69334 |
| 43.85524 | 10.18893 |
| 43.87556 | 10.97696 |
| 43.89588 | 9.99757 |
| 43.9162 | 11.47484 |
| 43.93652 | 11.77649 |
| 43.95684 | 14.46271 |
| 43.97716 | 18.27455 |
| 43.99748 | 18.23748 |
| 44.01781 | 17.28198 |
| 44.03813 | 15.35684 |
| 44.05845 | 23.59887 |
| 44.07877 | 26.62694 |
| 44.09909 | 30.64841 |
| 44.11941 | 31.34153 |
| 44.13973 | 31.1899 |
| 44.16005 | 34.5467 |
| 44.18038 | 37.20118 |
| 44.2007 | 40.28559 |
| 44.22102 | 41.33373 |
| 44.24134 | 40.16775 |
| 44.26166 | 45.60232 |
| 44.28198 | 46.50965 |
| 44.3023 | 49.71159 |
| 44.32262 | 48.29563 |
| 44.34294 | 45.96896 |
| 44.36327 | 41.42143 |
| 44.38359 | 37.27664 |
| 44.40391 | 35.13255 |
| 44.42423 | 34.21857 |
| 44.44455 | 34.44609 |
| 44.46487 | 31.3133 |
| 44.48519 | 31.77933 |
| 44.50551 | 30.98741 |
| 44.52584 | 32.3163 |
| 44.54616 | 33.79061 |
| 44.56648 | 30.9844 |
| 44.5868 | 28.53082 |
| 44.60712 | 28.2883 |
| 44.62744 | 25.0805 |
| 44.64776 | 21.82228 |
| 44.66808 | 18.12493 |
| 44.68841 | 15.81701 |
| 44.70873 | 14.53676 |
| 44.72905 | 12.55251 |
| 44.74937 | 14.68133 |
| 44.76969 | 12.17984 |
| 44.79001 | 14.76014 |
| 44.81033 | 11.33202 |
| 44.83065 | 12.19126 |
| 44.85097 | 14.31716 |
| 44.8713 | 13.6326 |
| 44.89162 | 10.35521 |
| 44.91194 | 10.52723 |
| 44.93226 | 14.00911 |
| 44.95258 | 15.04533 |
| 44.9729 | 15.36094 |
| 44.99322 | 15.58404 |
| 45.01354 | 12.30003 |
| 45.03387 | 16.41469 |
| 45.05419 | 18.33006 |
| 45.07451 | 21.33024 |
| 45.09483 | 17.74119 |
| 45.11515 | 15.45617 |
| 45.13547 | 15.66274 |
| 45.15579 | 18.78038 |
| 45.17611 | 19.40396 |
| 45.19644 | 22.47288 |
| 45.21676 | 21.3102 |
| 45.23708 | 22.84651 |
| 45.2574 | 24.77101 |
| 45.27772 | 26.42045 |
| 45.29804 | 19.87008 |
| 45.31836 | 19.51296 |
| 45.33868 | 17.65242 |
| 45.35901 | 18.50823 |
| 45.37933 | 15.97199 |
| 45.39965 | 16.70339 |
| 45.41997 | 16.54719 |
| 45.44029 | 18.60897 |
| 45.46061 | 20.97273 |
| 45.48093 | 21.08821 |
| 45.50125 | 20.27888 |
| 45.52157 | 20.46255 |
| 45.5419 | 21.0863 |
| 45.56222 | 16.95793 |
| 45.58254 | 8.4523 |
| 45.60286 | 7.63783 |
| 45.62318 | 6.92205 |
| 45.6435 | 9.34148 |
| 45.66382 | 12.11031 |
| 45.68414 | 16.56785 |
| 45.70447 | 16.38824 |
| 45.72479 | 16.02004 |
| 45.74511 | 16.95251 |
| 45.76543 | 20.10186 |
| 45.78575 | 21.29424 |
| 45.80607 | 21.21596 |
| 45.82639 | 20.34628 |
| 45.84671 | 23.03548 |
| 45.86704 | 24.71037 |
| 45.88736 | 24.92451 |
| 45.90768 | 27.87571 |
| 45.928 | 32.23664 |
| 45.94832 | 35.75195 |
| 45.96864 | 40.27062 |
| 45.98896 | 42.97375 |
| 46.00928 | 44.50349 |
| 46.02961 | 48.225 |
| 46.04993 | 52.03156 |
| 46.07025 | 51.66528 |
| 46.09057 | 51.09522 |
| 46.11089 | 54.46597 |
| 46.13121 | 57.81524 |
| 46.15153 | 61.28119 |
| 46.17185 | 57.84757 |
| 46.19217 | 60.48657 |
| 46.2125 | 59.30396 |
| 46.23282 | 55.60961 |
| 46.25314 | 57.5278 |
| 46.27346 | 55.15366 |
| 46.29378 | 54.83115 |
| 46.3141 | 52.4913 |
| 46.33442 | 54.07942 |
| 46.35474 | 54.51452 |
| 46.37507 | 50.83752 |
| 46.39539 | 49.5079 |
| 46.41571 | 51.44932 |
| 46.43603 | 47.39023 |
| 46.45635 | 46.852 |
| 46.47667 | 44.78217 |
| 46.49699 | 38.36347 |
| 46.51731 | 31.75157 |
| 46.53764 | 31.8567 |
| 46.55796 | 30.92465 |
| 46.57828 | 22.82684 |
| 46.5986 | 22.48748 |
| 46.61892 | 26.61726 |
| 46.63924 | 24.43064 |
| 46.65956 | 26.50949 |
| 46.67988 | 25.1619 |
| 46.70021 | 18.05506 |
| 46.72053 | 16.33691 |
| 46.74085 | 17.91631 |
| 46.76117 | 18.02804 |
| 46.78149 | 20.07782 |
| 46.80181 | 17.98758 |
| 46.82213 | 18.04578 |
| 46.84245 | 21.57816 |
| 46.86277 | 24.71393 |
| 46.8831 | 24.94273 |
| 46.90342 | 28.6221 |
| 46.92374 | 31.41467 |
| 46.94406 | 31.82505 |
| 46.96438 | 33.60708 |
| 46.9847 | 34.44542 |
| 47.00502 | 33.94028 |
| 47.02534 | 34.50913 |
| 47.04567 | 37.32911 |
| 47.06599 | 34.06502 |
| 47.08631 | 33.1098 |
| 47.10663 | 31.27083 |
| 47.12695 | 29.9667 |
| 47.14727 | 29.3324 |
| 47.16759 | 27.91986 |
| 47.18791 | 27.21608 |
| 47.20824 | 35.02416 |
| 47.22856 | 31.73641 |
| 47.24888 | 36.0769 |
| 47.2692 | 34.70942 |
| 47.28952 | 34.49095 |
| 47.30984 | 33.10139 |
| 47.33016 | 32.43363 |
| 47.35048 | 34.7423 |
| 47.37081 | 36.38675 |
| 47.39113 | 34.51867 |
| 47.41145 | 35.34883 |
| 47.43177 | 36.88509 |
| 47.45209 | 36.22522 |
| 47.47241 | 41.24279 |
| 47.49273 | 44.76999 |
| 47.51305 | 48.97311 |
| 47.53337 | 51.55697 |
| 47.5537 | 59.6524 |
| 47.57402 | 61.9427 |
| 47.59434 | 62.78063 |
| 47.61466 | 71.62981 |
| 47.63498 | 71.88667 |
| 47.6553 | 73.94238 |
| 47.67562 | 77.80598 |
| 47.69594 | 72.8958 |
| 47.71627 | 72.13736 |
| 47.73659 | 78.4741 |
| 47.75691 | 77.21349 |
| 47.77723 | 79.63435 |
| 47.79755 | 82.15631 |
| 47.81787 | 82.25607 |
| 47.83819 | 79.69648 |
| 47.85851 | 80.34423 |
| 47.87884 | 80.26107 |
| 47.89916 | 78.97879 |
| 47.91948 | 80.09815 |
| 47.9398 | 78.1293 |
| 47.96012 | 73.81585 |
| 47.98044 | 71.23398 |
| 48.00076 | 69.03395 |
| 48.02108 | 67.08684 |
| 48.0414 | 61.80774 |
| 48.06173 | 58.24024 |
| 48.08205 | 54.56866 |
| 48.10237 | 54.79375 |
| 48.12269 | 50.66525 |
| 48.14301 | 45.86207 |
| 48.16333 | 45.14645 |
| 48.18365 | 38.58296 |
| 48.20397 | 35.91902 |
| 48.2243 | 32.96811 |
| 48.24462 | 27.6971 |
| 48.26494 | 21.11438 |
| 48.28526 | 14.93822 |
| 48.30558 | 13.42667 |
| 48.3259 | 10.20971 |
| 48.34622 | 7.22621 |
| 48.36654 | 5.68207 |
| 48.38687 | 3.46625 |
| 48.40719 | 2.90563 |
| 48.42751 | 3.79022 |
| 48.44783 | 5.70822 |
| 48.46815 | 2.35573 |
| 48.48847 | 1.72659 |
| 48.50879 | 3.84049 |
| 48.52911 | -0.22827 |
| 48.54944 | -2.98124 |
| 48.56976 | -0.76964 |
| 48.59008 | -0.39951 |
| 48.6104 | -0.80762 |
| 48.63072 | -1.08839 |
| 48.65104 | -3.31115 |
| 48.67136 | -2.96073 |
| 48.69168 | -0.0994 |
| 48.712 | -1.70647 |
| 48.73233 | 2.56984 |
| 48.75265 | 4.42289 |
| 48.77297 | 8.38713 |
| 48.79329 | 12.49377 |
| 48.81361 | 10.39887 |
| 48.83393 | 13.47188 |
| 48.85425 | 14.06327 |
| 48.87457 | 22.86639 |
| 48.8949 | 24.22472 |
| 48.91522 | 25.59032 |
| 48.93554 | 29.09847 |
| 48.95586 | 34.01621 |
| 48.97618 | 35.2798 |
| 48.9965 | 38.53218 |
| 49.01682 | 41.51172 |
| 49.03714 | 43.1792 |
| 49.05747 | 41.94912 |
| 49.07779 | 43.58582 |
| 49.09811 | 45.5246 |
| 49.11843 | 48.72133 |
| 49.13875 | 53.05528 |
| 49.15907 | 56.48635 |
| 49.17939 | 59.58986 |
| 49.19971 | 63.16268 |
| 49.22004 | 69.18947 |
| 49.24036 | 69.07094 |
| 49.26068 | 69.58621 |
| 49.281 | 71.71152 |
| 49.30132 | 71.63594 |
| 49.32164 | 71.03562 |
| 49.34196 | 70.62496 |
| 49.36228 | 67.95117 |
| 49.3826 | 71.23458 |
| 49.40293 | 68.6437 |
| 49.42325 | 65.16212 |
| 49.44357 | 61.844 |
| 49.46389 | 60.54716 |
| 49.48421 | 57.11637 |
| 49.50453 | 56.08946 |
| 49.52485 | 52.95626 |
| 49.54517 | 46.45333 |
| 49.5655 | 50.39912 |
| 49.58582 | 46.52505 |
| 49.60614 | 44.83138 |
| 49.62646 | 42.81589 |
| 49.64678 | 42.03697 |
| 49.6671 | 42.38364 |
| 49.68742 | 39.26101 |
| 49.70774 | 42.48542 |
| 49.72807 | 38.63776 |
| 49.74839 | 39.41685 |
| 49.76871 | 39.18928 |
| 49.78903 | 37.48687 |
| 49.80935 | 36.65306 |
| 49.82967 | 36.54993 |
| 49.84999 | 38.47242 |
| 49.87031 | 37.75906 |
| 49.89064 | 36.00424 |
| 49.91096 | 31.72527 |
| 49.93128 | 31.25707 |
| 49.9516 | 32.32462 |
| 49.97192 | 33.32317 |
| 49.99224 | 29.844 |
| 50.01256 | 29.75186 |
| 50.03288 | 32.2898 |
| 50.0532 | 36.1554 |
| 50.07353 | 36.95426 |
| 50.09385 | 44.26576 |
| 50.11417 | 48.9717 |
| 50.13449 | 52.88437 |
| 50.15481 | 53.76659 |
| 50.17513 | 50.68811 |
| 50.19545 | 49.26493 |
| 50.21577 | 48.91993 |
| 50.2361 | 48.23881 |
| 50.25642 | 48.67735 |
| 50.27674 | 49.61878 |
| 50.29706 | 44.68608 |
| 50.31738 | 43.51707 |
| 50.3377 | 41.82742 |
| 50.35802 | 39.54421 |
| 50.37834 | 38.44279 |
| 50.39867 | 37.72634 |
| 50.41899 | 34.98191 |
| 50.43931 | 30.70013 |
| 50.45963 | 30.52393 |
| 50.47995 | 27.47175 |
| 50.50027 | 22.65553 |
| 50.52059 | 20.29938 |
| 50.54091 | 21.61285 |
| 50.56123 | 23.4575 |
| 50.58156 | 27.30789 |
| 50.60188 | 23.31942 |
| 50.6222 | 22.17252 |
| 50.64252 | 26.73954 |
| 50.66284 | 28.78472 |
| 50.68316 | 28.20459 |
| 50.70348 | 29.17251 |
| 50.7238 | 30.90752 |
| 50.74413 | 29.89153 |
| 50.76445 | 30.77291 |
| 50.78477 | 29.52101 |
| 50.80509 | 30.24081 |
| 50.82541 | 33.86732 |
| 50.84573 | 27.92532 |
| 50.86605 | 26.64595 |
| 50.88637 | 25.72469 |
| 50.9067 | 20.29351 |
| 50.92702 | 21.53184 |
| 50.94734 | 23.08009 |
| 50.96766 | 22.89609 |
| 50.98798 | 25.79923 |
| 51.0083 | 25.94477 |
| 51.02862 | 28.55865 |
| 51.04894 | 30.27139 |
| 51.06927 | 28.8653 |
| 51.08959 | 23.5014 |
| 51.10991 | 21.75157 |
| 51.13023 | 20.93354 |
| 51.15055 | 18.07241 |
| 51.17087 | 15.18182 |
| 51.19119 | 14.07971 |
| 51.21151 | 15.03475 |
| 51.23183 | 14.42897 |
| 51.25216 | 16.40041 |
| 51.27248 | 15.95894 |
| 51.2928 | 16.01676 |
| 51.31312 | 14.49184 |
| 51.33344 | 15.44092 |
| 51.35376 | 10.56189 |
| 51.37408 | 13.33118 |
| 51.3944 | 9.86424 |
| 51.41473 | 8.02806 |
| 51.43505 | 7.99979 |
| 51.45537 | 5.49962 |
| 51.47569 | 9.00975 |
| 51.49601 | 10.11709 |
| 51.51633 | 12.52231 |
| 51.53665 | 18.01277 |
| 51.55697 | 16.7842 |
| 51.5773 | 16.85444 |
| 51.59762 | 18.49149 |
| 51.61794 | 15.2021 |
| 51.63826 | 20.26746 |
| 51.65858 | 24.75578 |
| 51.6789 | 23.82778 |
| 51.69922 | 22.14224 |
| 51.71954 | 23.5885 |
| 51.73987 | 21.02283 |
| 51.76019 | 24.10546 |
| 51.78051 | 23.07788 |
| 51.80083 | 23.12322 |
| 51.82115 | 24.02836 |
| 51.84147 | 25.58923 |
| 51.86179 | 22.44491 |
| 51.88211 | 23.20874 |
| 51.90243 | 21.88717 |
| 51.92276 | 21.48752 |
| 51.94308 | 22.34732 |
| 51.9634 | 26.02719 |
| 51.98372 | 26.8242 |
| 52.00404 | 29.77968 |
| 52.02436 | 28.08308 |
| 52.04468 | 29.5346 |
| 52.065 | 31.06395 |
| 52.08533 | 31.5255 |
| 52.10565 | 31.44746 |
| 52.12597 | 33.67182 |
| 52.14629 | 37.05133 |
| 52.16661 | 35.65202 |
| 52.18693 | 38.61273 |
| 52.20725 | 43.58732 |
| 52.22757 | 44.17204 |
| 52.2479 | 44.76904 |
| 52.26822 | 48.42846 |
| 52.28854 | 49.10374 |
| 52.30886 | 46.44903 |
| 52.32918 | 39.71392 |
| 52.3495 | 37.27779 |
| 52.36982 | 36.79879 |
| 52.39014 | 35.83545 |
| 52.41047 | 31.86156 |
| 52.43079 | 33.62794 |
| 52.45111 | 34.93494 |
| 52.47143 | 32.03344 |
| 52.49175 | 31.5501 |
| 52.51207 | 32.49919 |
| 52.53239 | 31.75291 |
| 52.55271 | 29.28725 |
| 52.57303 | 22.85575 |
| 52.59336 | 24.75881 |
| 52.61368 | 24.81638 |
| 52.634 | 24.97959 |
| 52.67464 | 23.81888 |
| 52.69496 | 18.40655 |
| 52.71528 | 17.8178 |
| 52.7356 | 15.24661 |
| 52.75593 | 14.23372 |
| 52.77625 | 15.43647 |
| 52.79657 | 14.27131 |
| 52.81689 | 11.80825 |
| 52.83721 | 12.36022 |
| 52.85753 | 9.83173 |
| 52.87785 | 8.35175 |
| 52.89817 | 5.98008 |
| 52.9185 | 3.92013 |
| 52.93882 | 4.46552 |
| 52.95914 | 6.01373 |
| 52.97946 | 6.26072 |
| 52.99978 | 8.60995 |
| 53.0201 | 7.44768 |
| 53.04042 | 10.89843 |
| 53.06074 | 11.25963 |
| 53.08106 | 13.61086 |
| 53.10139 | 13.63265 |
| 53.12171 | 13.2496 |
| 53.14203 | 11.81116 |
| 53.16235 | 11.40162 |
| 53.18267 | 14.01896 |
| 53.20299 | 17.35236 |
| 53.22331 | 18.67828 |
| 53.24363 | 18.11677 |
| 53.26396 | 14.83494 |
| 53.28428 | 15.41207 |
| 53.3046 | 16.24513 |
| 53.32492 | 15.89963 |
| 53.34524 | 12.86589 |
| 53.36556 | 14.40521 |
| 53.38588 | 16.15599 |
| 53.4062 | 15.77205 |
| 53.42653 | 15.23122 |
| 53.44685 | 15.44358 |
| 53.46717 | 14.33591 |
| 53.48749 | 13.24887 |
| 53.50781 | 11.49364 |
| 53.52813 | 14.51065 |
| 53.54845 | 17.49409 |
| 53.56877 | 16.82983 |
| 53.5891 | 16.66856 |
| 53.60942 | 17.07912 |
| 53.62974 | 20.43912 |
| 53.65006 | 21.03041 |
| 53.67038 | 21.6562 |
| 53.6907 | 21.72755 |
| 53.71102 | 21.96157 |
| 53.73134 | 25.97235 |
| 53.75166 | 29.03158 |
| 53.77199 | 28.6377 |
| 53.79231 | 33.7832 |
| 53.81263 | 33.2533 |
| 53.83295 | 36.23846 |
| 53.85327 | 38.61875 |
| 53.87359 | 38.3429 |
| 53.89391 | 36.98716 |
| 53.91423 | 39.99873 |
| 53.93456 | 39.3704 |
| 53.95488 | 42.20151 |
| 53.9752 | 41.97934 |
| 53.99552 | 42.65727 |
| 54.01584 | 41.49142 |
| 54.03616 | 42.92579 |
| 54.05648 | 42.62506 |
| 54.0768 | 36.41356 |
| 54.09713 | 35.87366 |
| 54.11745 | 36.89582 |
| 54.13777 | 37.71081 |
| 54.15809 | 39.02688 |
| 54.17841 | 41.9163 |
| 54.19873 | 43.71329 |
| 54.21905 | 44.75555 |
| 54.23937 | 40.32813 |
| 54.2597 | 37.0905 |
| 54.28002 | 37.49525 |
| 54.30034 | 40.33892 |
| 54.32066 | 42.78021 |
| 54.34098 | 42.29968 |
| 54.3613 | 41.02375 |
| 54.38162 | 41.09899 |
| 54.40194 | 41.35117 |
| 54.42226 | 38.63049 |
| 54.44259 | 38.20703 |
| 54.46291 | 37.93645 |
| 54.48323 | 36.70681 |
| 54.50355 | 36.69843 |
| 54.52387 | 40.81737 |
| 54.54419 | 40.93046 |
| 54.56451 | 41.86384 |
| 54.58483 | 40.56532 |
| 54.60516 | 41.1684 |
| 54.62548 | 41.95273 |
| 54.6458 | 40.65687 |
| 54.66612 | 39.28786 |
| 54.68644 | 39.20223 |
| 54.70676 | 37.19014 |
| 54.72708 | 34.19249 |
| 54.7474 | 35.88751 |
| 54.76773 | 35.56451 |
| 54.78805 | 32.25108 |
| 54.80837 | 32.40715 |
| 54.82869 | 30.58023 |
| 54.84901 | 30.54142 |
| 54.86933 | 31.34073 |
| 54.88965 | 29.85915 |
| 54.90997 | 31.02021 |
| 54.9303 | 32.61344 |
| 54.95062 | 31.20475 |
| 54.97094 | 29.83292 |
| 54.99126 | 26.59349 |
| 55.01158 | 30.0925 |
| 55.0319 | 28.96888 |
| 55.05222 | 31.01235 |
| 55.07254 | 27.29496 |
| 55.09286 | 30.40314 |
| 55.11319 | 34.21737 |
| 55.13351 | 34.06777 |
| 55.15383 | 34.95307 |
| 55.17415 | 36.2626 |
| 55.19447 | 35.28165 |
| 55.21479 | 32.38078 |
| 55.23511 | 33.68174 |
| 55.25543 | 30.77344 |
| 55.27576 | 31.9428 |
| 55.29608 | 36.62398 |
| 55.3164 | 42.96552 |
| 55.33672 | 44.25241 |
| 55.35704 | 44.73138 |
| 55.37736 | 46.22487 |
| 55.39768 | 46.16452 |
| 55.418 | 47.70236 |
| 55.43833 | 47.60161 |
| 55.45865 | 51.43859 |
| 55.47897 | 47.21119 |
| 55.49929 | 49.20828 |
| 55.51961 | 49.42038 |
| 55.53993 | 46.94462 |
| 55.56025 | 46.40213 |
| 55.58057 | 46.99765 |
| 55.6009 | 46.69554 |
| 55.62122 | 48.00638 |
| 55.64154 | 46.27749 |
| 55.66186 | 41.50205 |
| 55.68218 | 41.08543 |
| 55.7025 | 40.63453 |
| 55.72282 | 40.27164 |
| 55.74314 | 39.11132 |
| 55.76346 | 39.36245 |
| 55.78379 | 40.35885 |
| 55.80411 | 39.40101 |
| 55.82443 | 41.49717 |
| 55.84475 | 45.06721 |
| 55.86507 | 45.92461 |
| 55.88539 | 46.38038 |
| 55.90571 | 47.23815 |
| 55.92603 | 48.86642 |
| 55.94636 | 47.9489 |
| 55.96668 | 46.6059 |
| 55.987 | 42.82194 |
| 56.00732 | 41.68251 |
| 56.02764 | 44.15771 |
| 56.04796 | 39.61876 |
| 56.06828 | 38.33878 |
| 56.0886 | 36.17747 |
| 56.10893 | 38.81761 |
| 56.12925 | 35.32869 |
| 56.14957 | 34.27691 |
| 56.16989 | 36.21063 |
| 56.19021 | 33.73327 |
| 56.21053 | 35.54173 |
| 56.23085 | 36.92332 |
| 56.25117 | 38.46466 |
| 56.27149 | 39.38633 |
| 56.29182 | 44.18355 |
| 56.31214 | 46.38184 |
| 56.33246 | 48.94503 |
| 56.35278 | 46.52506 |
| 56.3731 | 45.65026 |
| 56.39342 | 46.06534 |
| 56.41374 | 44.87328 |
| 56.43406 | 45.45035 |
| 56.45439 | 46.32219 |
| 56.47471 | 45.75037 |
| 56.49503 | 45.47261 |
| 56.51535 | 50.34631 |
| 56.53567 | 49.83644 |
| 56.55599 | 49.49691 |
| 56.57631 | 54.00344 |
| 56.59663 | 53.50235 |
| 56.61696 | 53.54526 |
| 56.63728 | 53.15751 |
| 56.6576 | 55.56633 |
| 56.67792 | 54.62297 |
| 56.69824 | 57.76224 |
| 56.71856 | 55.80339 |
| 56.73888 | 53.05091 |
| 56.7592 | 53.24734 |
| 56.77953 | 52.50378 |
| 56.79985 | 55.22462 |
| 56.82017 | 55.22305 |
| 56.84049 | 54.87239 |
| 56.86081 | 54.09085 |
| 56.88113 | 51.38234 |
| 56.90145 | 48.45605 |
| 56.92177 | 50.33314 |
| 56.94209 | 47.10442 |
| 56.96242 | 39.76694 |
| 56.98274 | 36.50276 |
| 57.00306 | 36.23074 |
| 57.02338 | 33.59966 |
| 57.0437 | 29.46932 |
| 57.06402 | 24.04832 |
| 57.08434 | 24.65299 |
| 57.10466 | 23.84713 |
| 57.12499 | 23.57346 |
| 57.14531 | 21.05523 |
| 57.16563 | 17.35405 |
| 57.18595 | 15.29171 |
| 57.20627 | 13.6693 |
| 57.22659 | 15.59468 |
| 57.24691 | 11.001 |
| 57.26723 | 10.64547 |
| 57.28756 | 10.63465 |
| 57.30788 | 9.77417 |
| 57.3282 | 6.86283 |
| 57.34852 | 4.02978 |
| 57.36884 | 3.73892 |
| 57.38916 | 1.95209 |
| 57.40948 | 3.87935 |
| 57.4298 | 4.4284 |
| 57.45013 | 8.85593 |
| 57.47045 | 12.40737 |
| 57.49077 | 15.93526 |
| 57.51109 | 17.15344 |
| 57.53141 | 16.76794 |
| 57.55173 | 17.8685 |
| 57.57205 | 17.82454 |
| 57.59237 | 19.36867 |
| 57.61269 | 21.75955 |
| 57.63302 | 24.29575 |
| 57.65334 | 27.36373 |
| 57.67366 | 26.53511 |
| 57.69398 | 24.92143 |
| 57.7143 | 27.9317 |
| 57.73462 | 27.30347 |
| 57.75494 | 30.06708 |
| 57.77526 | 35.22163 |
| 57.79559 | 34.05008 |
| 57.81591 | 36.11352 |
| 57.83623 | 35.06279 |
| 57.85655 | 37.52549 |
| 57.87687 | 37.11942 |
| 57.89719 | 40.69935 |
| 57.91751 | 41.77887 |
| 57.93783 | 45.1885 |
| 57.95816 | 42.47461 |
| 57.97848 | 42.93577 |
| 57.9988 | 44.89858 |
| 58.01912 | 43.97001 |
| 58.03944 | 42.66001 |
| 58.05976 | 40.76884 |
| 58.08008 | 41.98405 |
| 58.1004 | 42.61635 |
| 58.12073 | 41.34231 |
| 58.14105 | 42.55437 |
| 58.16137 | 41.35755 |
| 58.18169 | 40.75083 |
| 58.20201 | 42.9078 |
| 58.22233 | 45.92652 |
| 58.24265 | 45.26106 |
| 58.26297 | 44.07259 |
| 58.28329 | 45.63549 |
| 58.30362 | 44.71347 |
| 58.32394 | 46.39227 |
| 58.34426 | 41.89713 |
| 58.36458 | 40.02984 |
| 58.3849 | 40.87066 |
| 58.40522 | 39.12321 |
| 58.42554 | 40.19062 |
| 58.44586 | 38.83203 |
| 58.46619 | 38.26183 |
| 58.48651 | 37.86713 |
| 58.50683 | 35.17271 |
| 58.52715 | 31.13741 |
| 58.54747 | 29.72127 |
| 58.56779 | 28.25836 |
| 58.58811 | 28.81739 |
| 58.60843 | 28.07534 |
| 58.62876 | 25.79057 |
| 58.64908 | 25.43702 |
| 58.6694 | 24.36622 |
| 58.68972 | 24.88017 |
| 58.71004 | 27.86953 |
| 58.73036 | 30.35354 |
| 58.75068 | 28.14345 |
| 58.771 | 26.71803 |
| 58.79132 | 24.53459 |
| 58.81165 | 23.48404 |
| 58.83197 | 23.53334 |
| 58.85229 | 25.80419 |
| 58.87261 | 26.72078 |
| 58.89293 | 23.7592 |
| 58.91325 | 25.43248 |
| 58.93357 | 23.10536 |
| 58.95389 | 22.18977 |
| 58.97422 | 22.02843 |
| 58.99454 | 22.32807 |
| 59.01486 | 22.49821 |
| 59.03518 | 22.97854 |
| 59.0555 | 24.41964 |
| 59.07582 | 23.7979 |
| 59.09614 | 24.78979 |
| 59.11646 | 26.83407 |
| 59.13679 | 29.5602 |
| 59.15711 | 32.54217 |
| 59.17743 | 33.76866 |
| 59.19775 | 31.64373 |
| 59.21807 | 31.69032 |
| 59.23839 | 28.96905 |
| 59.25871 | 28.59176 |
| 59.27903 | 28.33599 |
| 59.29936 | 25.49369 |
| 59.31968 | 25.70716 |
| 59.34 | 23.86148 |
| 59.36032 | 23.77648 |
| 59.38064 | 27.78272 |
| 59.40096 | 29.16797 |
| 59.42128 | 30.15247 |
| 59.4416 | 30.85859 |
| 59.46192 | 27.1798 |
| 59.48225 | 30.72117 |
| 59.50257 | 32.30134 |
| 59.52289 | 32.93711 |
| 59.54321 | 33.33908 |
| 59.56353 | 32.05154 |
| 59.58385 | 33.99582 |
| 59.60417 | 38.1307 |
| 59.62449 | 38.50049 |
| 59.64482 | 39.83375 |
| 59.66514 | 40.36198 |
| 59.68546 | 40.20978 |
| 59.70578 | 38.61569 |
| 59.7261 | 36.21894 |
| 59.74642 | 34.12696 |
| 59.76674 | 32.30378 |
| 59.78706 | 30.20762 |
| 59.80739 | 29.14842 |
| 59.82771 | 28.64896 |
| 59.84803 | 26.26709 |
| 59.86835 | 27.92962 |
| 59.88867 | 28.4933 |
| 59.90899 | 27.76543 |
| 59.92931 | 26.33269 |
| 59.94963 | 26.17749 |
| 59.96996 | 27.76377 |
| 59.99028 | 28.18867 |
| 60.0106 | 25.33937 |
| 60.03092 | 26.68766 |
| 60.05124 | 23.87597 |
| 60.07156 | 21.22901 |
| 60.09188 | 17.35269 |
| 60.1122 | 14.62852 |
| 60.13252 | 14.07989 |
| 60.15285 | 12.7634 |
| 60.17317 | 11.2141 |
| 60.19349 | 8.51739 |
| 60.21381 | 4.75735 |
| 60.23413 | 6.05421 |
| 60.25445 | 2.20595 |
| 60.27477 | 4.85138 |
| 60.29509 | 3.95453 |
| 60.31542 | 6.78311 |
| 60.33574 | 10.22337 |
| 60.35606 | 9.48543 |
| 60.37638 | 12.76947 |
| 60.3967 | 14.58339 |
| 60.41702 | 12.64264 |
| 60.43734 | 13.00737 |
| 60.45766 | 13.16696 |
| 60.47799 | 13.29694 |
| 60.49831 | 17.40463 |
| 60.51863 | 16.16455 |
| 60.53895 | 13.25696 |
| 60.55927 | 14.65274 |
| 60.57959 | 15.67001 |
| 60.59991 | 14.72684 |
| 60.62023 | 14.28713 |
| 60.64056 | 13.14451 |
| 60.66088 | 14.03538 |
| 60.6812 | 12.35122 |
| 60.70152 | 13.79499 |
| 60.72184 | 12.96813 |
| 60.74216 | 16.24155 |
| 60.76248 | 17.79064 |
| 60.7828 | 18.50374 |
| 60.80312 | 19.27304 |
| 60.82345 | 23.0286 |
| 60.84377 | 24.71185 |
| 60.86409 | 27.13348 |
| 60.88441 | 25.69858 |
| 60.90473 | 25.20612 |
| 60.92505 | 21.22245 |
| 60.94537 | 20.97059 |
| 60.96569 | 22.57915 |
| 60.98602 | 21.33089 |
| 61.00634 | 16.7703 |
| 61.02666 | 17.44772 |
| 61.04698 | 20.08651 |
| 61.0673 | 17.59407 |
| 61.08762 | 13.89732 |
| 61.10794 | 17.25622 |
| 61.12826 | 18.33851 |
| 61.14859 | 17.64609 |
| 61.16891 | 18.59511 |
| 61.18923 | 17.85116 |
| 61.20955 | 21.93148 |
| 61.22987 | 22.26983 |
| 61.25019 | 24.3517 |
| 61.27051 | 24.47214 |
| 61.29083 | 23.70384 |
| 61.31115 | 24.83486 |
| 61.33148 | 26.04429 |
| 61.3518 | 24.52595 |
| 61.37212 | 24.23755 |
| 61.39244 | 26.36664 |
| 61.41276 | 29.76754 |
| 61.43308 | 27.39863 |
| 61.4534 | 29.85491 |
| 61.47372 | 29.73077 |
| 61.49405 | 28.15827 |
| 61.51437 | 29.30374 |
| 61.53469 | 29.56186 |
| 61.55501 | 28.82821 |
| 61.57533 | 35.08301 |
| 61.59565 | 33.78164 |
| 61.61597 | 33.90752 |
| 61.63629 | 38.82855 |
| 61.65662 | 42.24515 |
| 61.67694 | 42.96065 |
| 61.69726 | 41.37641 |
| 61.71758 | 44.10481 |
| 61.7379 | 47.63814 |
| 61.75822 | 50.80447 |
| 61.77854 | 48.26298 |
| 61.79886 | 49.96044 |
| 61.81919 | 50.547 |
| 61.83951 | 53.26777 |
| 61.85983 | 51.44061 |
| 61.88015 | 49.90705 |
| 61.90047 | 50.15635 |
| 61.92079 | 50.64804 |
| 61.94111 | 49.57751 |
| 61.96143 | 50.68506 |
| 61.98175 | 50.48993 |
| 62.00208 | 52.92314 |
| 62.0224 | 55.40747 |
| 62.04272 | 56.71162 |
| 62.06304 | 55.57282 |
| 62.08336 | 54.94739 |
| 62.10368 | 53.00379 |
| 62.124 | 55.68281 |
| 62.14432 | 53.38804 |
| 62.16465 | 52.57898 |
| 62.18497 | 54.07713 |
| 62.20529 | 50.98788 |
| 62.22561 | 53.21608 |
| 62.24593 | 50.90961 |
| 62.26625 | 50.49941 |
| 62.28657 | 48.09658 |
| 62.30689 | 46.21624 |
| 62.32722 | 47.86704 |
| 62.34754 | 46.44111 |
| 62.36786 | 43.43472 |
| 62.38818 | 40.05357 |
| 62.4085 | 38.29789 |
| 62.42882 | 35.81247 |
| 62.44914 | 34.64581 |
| 62.46946 | 35.22417 |
| 62.48979 | 32.96397 |
| 62.51011 | 31.56358 |
| 62.53043 | 29.84266 |
| 62.55075 | 28.13438 |
| 62.57107 | 27.33773 |
| 62.59139 | 27.16458 |
| 62.61171 | 25.18858 |
| 62.63203 | 22.21109 |
| 62.65235 | 21.46886 |
| 62.67268 | 22.41803 |
| 62.693 | 20.41726 |
| 62.71332 | 21.01434 |
| 62.73364 | 21.71678 |
| 62.75396 | 21.18841 |
| 62.77428 | 18.91617 |
| 62.7946 | 18.53162 |
| 62.81492 | 18.53029 |
| 62.83525 | 18.1289 |
| 62.85557 | 19.52749 |
| 62.87589 | 16.93904 |
| 62.89621 | 15.02526 |
| 62.91653 | 15.69784 |
| 62.93685 | 13.75573 |
| 62.95717 | 13.44794 |
| 62.97749 | 13.30307 |
| 62.99782 | 15.13701 |
| 63.01814 | 14.89663 |
| 63.03846 | 9.74948 |
| 63.05878 | 5.18241 |
| 63.0791 | 7.80777 |
| 63.09942 | 10.17806 |
| 63.11974 | 8.23563 |
| 63.14006 | 10.23161 |
| 63.16039 | 12.78743 |
| 63.18071 | 13.82555 |
| 63.20103 | 14.97342 |
| 63.22135 | 13.13444 |
| 63.24167 | 17.32127 |
| 63.26199 | 18.29699 |
| 63.28231 | 17.69514 |
| 63.30263 | 18.45914 |
| 63.32295 | 17.16603 |
| 63.34328 | 14.83687 |
| 63.3636 | 13.66695 |
| 63.38392 | 12.46844 |
| 63.40424 | 13.27241 |
| 63.42456 | 15.93706 |
| 63.44488 | 16.31617 |
| 63.4652 | 18.92738 |
| 63.48552 | 15.61264 |
| 63.50585 | 16.0899 |
| 63.52617 | 15.25102 |
| 63.54649 | 14.11479 |
| 63.56681 | 17.50712 |
| 63.58713 | 18.30462 |
| 63.60745 | 21.20885 |
| 63.62777 | 23.28542 |
| 63.64809 | 23.63327 |
| 63.66842 | 25.91316 |
| 63.68874 | 26.9821 |
| 63.70906 | 25.11295 |
| 63.72938 | 23.65118 |
| 63.7497 | 24.41699 |
| 63.77002 | 21.89403 |
| 63.79034 | 21.28331 |
| 63.81066 | 22.58867 |
| 63.83099 | 21.12822 |
| 63.85131 | 21.63704 |
| 63.87163 | 21.96625 |
| 63.89195 | 21.56521 |
| 63.91227 | 19.96117 |
| 63.93259 | 17.28755 |
| 63.95291 | 14.67638 |
| 63.97323 | 14.66 |
| 63.99355 | 15.62226 |
| 64.01388 | 16.71987 |
| 64.0342 | 15.10926 |
| 64.05452 | 14.82856 |
| 64.07484 | 16.4644 |
| 64.09516 | 19.88629 |
| 64.11548 | 18.96521 |
| 64.1358 | 17.54131 |
| 64.15612 | 17.7836 |
| 64.17645 | 14.4045 |
| 64.19677 | 14.26713 |
| 64.21709 | 13.86154 |
| 64.23741 | 14.80609 |
| 64.25773 | 17.54947 |
| 64.27805 | 15.58348 |
| 64.29837 | 14.83259 |
| 64.31869 | 15.05895 |
| 64.33902 | 14.37779 |
| 64.35934 | 10.62052 |
| 64.37966 | 11.99162 |
| 64.39998 | 14.22869 |
| 64.4203 | 13.06321 |
| 64.44062 | 12.40874 |
| 64.46094 | 12.23376 |
| 64.48126 | 13.56388 |
| 64.50158 | 16.8231 |
| 64.52191 | 20.26257 |
| 64.54223 | 19.52971 |
| 64.56255 | 22.32731 |
| 64.58287 | 24.27358 |
| 64.60319 | 24.73159 |
| 64.62351 | 26.1564 |
| 64.64383 | 24.30804 |
| 64.66415 | 22.77367 |
| 64.68448 | 22.1347 |
| 64.7048 | 19.0214 |
| 64.72512 | 18.93875 |
| 64.74544 | 18.44361 |
| 64.76576 | 19.24359 |
| 64.78608 | 14.29096 |
| 64.8064 | 17.51738 |
| 64.82672 | 17.99688 |
| 64.86737 | 14.15025 |
| 64.88769 | 15.87971 |
| 64.90801 | 16.62395 |
| 64.92833 | 14.13872 |
| 64.94865 | 14.66584 |
| 64.96897 | 14.87316 |
| 64.98929 | 18.86109 |
| 65.00962 | 19.38915 |
| 65.02994 | 16.84145 |
| 65.05026 | 16.4274 |
| 65.07058 | 16.81898 |
| 65.0909 | 16.1869 |
| 65.11122 | 16.79553 |
| 65.13154 | 16.25431 |
| 65.15186 | 14.41011 |
| 65.17218 | 15.83538 |
| 65.19251 | 15.76192 |
| 65.21283 | 15.51321 |
| 65.23315 | 18.92328 |
| 65.25347 | 18.32037 |
| 65.27379 | 18.45927 |
| 65.29411 | 17.30436 |
| 65.31443 | 17.03754 |
| 65.33475 | 18.80674 |
| 65.35508 | 19.14481 |
| 65.3754 | 15.48793 |
| 65.39572 | 14.04028 |
| 65.41604 | 19.25044 |
| 65.43636 | 18.55002 |
| 65.45668 | 17.49067 |
| 65.477 | 18.65636 |
| 65.49732 | 22.05385 |
| 65.51765 | 23.96708 |
| 65.53797 | 22.18556 |
| 65.55829 | 18.78906 |
| 65.57861 | 18.39193 |
| 65.59893 | 20.03966 |
| 65.61925 | 18.37129 |
| 65.63957 | 17.24036 |
| 65.65989 | 16.69553 |
| 65.68022 | 15.11162 |
| 65.70054 | 14.04018 |
| 65.72086 | 13.3813 |
| 65.74118 | 13.16576 |
| 65.7615 | 14.89869 |
| 65.78182 | 13.7564 |
| 65.80214 | 11.8653 |
| 65.82246 | 11.27888 |
| 65.84278 | 13.32061 |
| 65.86311 | 13.23134 |
| 65.88343 | 13.14657 |
| 65.90375 | 16.10348 |
| 65.92407 | 16.1418 |
| 65.94439 | 18.05843 |
| 65.96471 | 19.00144 |
| 65.98503 | 16.79567 |
| 66.00535 | 14.32452 |
| 66.02568 | 12.58211 |
| 66.046 | 13.92363 |
| 66.06632 | 14.81127 |
| 66.08664 | 14.93221 |
| 66.10696 | 13.5025 |
| 66.12728 | 13.08367 |
| 66.1476 | 12.4947 |
| 66.16792 | 11.96661 |
| 66.18825 | 10.03912 |
| 66.20857 | 8.06352 |
| 66.22889 | 9.25348 |
| 66.24921 | 8.9742 |
| 66.26953 | 9.37681 |
| 66.28985 | 9.31787 |
| 66.31017 | 10.24476 |
| 66.33049 | 8.26404 |
| 66.35082 | 8.82747 |
| 66.37114 | 9.23326 |
| 66.39146 | 6.76899 |
| 66.41178 | 8.43867 |
| 66.4321 | 10.01136 |
| 66.45242 | 10.48568 |
| 66.47274 | 8.71436 |
| 66.49306 | 8.58239 |
| 66.51338 | 7.20183 |
| 66.53371 | 3.65349 |
| 66.55403 | -1.66807 |
| 66.57435 | -0.07003 |
| 66.59467 | -0.48942 |
| 66.61499 | 2.39437 |
| 66.63531 | 2.34788 |
| 66.65563 | 3.94198 |
| 66.67595 | 3.40103 |
| 66.69628 | 4.05261 |
| 66.7166 | 5.87427 |
| 66.73692 | 9.2923 |
| 66.75724 | 12.23368 |
| 66.77756 | 10.43444 |
| 66.79788 | 10.16558 |
| 66.8182 | 9.74417 |
| 66.83852 | 8.42431 |
| 66.85885 | 7.34046 |
| 66.87917 | 7.87115 |
| 66.89949 | 6.47361 |
| 66.91981 | 6.73301 |
| 66.94013 | 6.87353 |
| 66.96045 | 3.66976 |
| 66.98077 | 2.11366 |
| 67.00109 | 3.40143 |
| 67.02141 | 3.21815 |
| 67.04174 | 5.33131 |
| 67.06206 | 4.58654 |
| 67.08238 | 3.00837 |
| 67.1027 | 5.62479 |
| 67.12302 | 7.01735 |
| 67.14334 | 6.92217 |
| 67.16366 | 7.42607 |
| 67.18398 | 7.49461 |
| 67.20431 | 9.00825 |
| 67.22463 | 14.62257 |
| 67.24495 | 16.52199 |
| 67.26527 | 15.60349 |
| 67.28559 | 15.20827 |
| 67.30591 | 18.36857 |
| 67.32623 | 17.45707 |
| 67.34655 | 17.88815 |
| 67.36688 | 21.10491 |
| 67.3872 | 21.63792 |
| 67.40752 | 19.58052 |
| 67.42784 | 19.77903 |
| 67.44816 | 20.57999 |
| 67.46848 | 18.74912 |
| 67.4888 | 14.90118 |
| 67.50912 | 12.54385 |
| 67.52945 | 12.3143 |
| 67.54977 | 14.13712 |
| 67.57009 | 14.98961 |
| 67.59041 | 15.02579 |
| 67.61073 | 12.61916 |
| 67.63105 | 17.2705 |
| 67.65137 | 12.93258 |
| 67.67169 | 12.35406 |
| 67.69201 | 11.81436 |
| 67.71234 | 12.98988 |
| 67.73266 | 13.11295 |
| 67.75298 | 11.36454 |
| 67.7733 | 9.21627 |
| 67.79362 | 3.02096 |
| 67.81394 | 2.91453 |
| 67.83426 | 1.49162 |
| 67.85458 | -0.24661 |
| 67.87491 | 1.17626 |
| 67.89523 | 1.49198 |
| 67.91555 | -1.15702 |
| 67.93587 | 1.62925 |
| 67.95619 | 5.22034 |
| 67.97651 | 6.90431 |
| 67.99683 | 6.32942 |
| 68.01715 | 8.16214 |
| 68.03748 | 7.00146 |
| 68.0578 | 6.52404 |
| 68.07812 | 7.85335 |
| 68.09844 | 7.40248 |
| 68.11876 | 9.32757 |
| 68.13908 | 6.29236 |
| 68.1594 | 6.63227 |
| 68.17972 | 7.0709 |
| 68.20005 | 6.65853 |
| 68.22037 | 7.3087 |
| 68.24069 | 9.11247 |
| 68.26101 | 7.61925 |
| 68.28133 | 5.13782 |
| 68.30165 | 7.27988 |
| 68.32197 | 11.69045 |
| 68.34229 | 11.81675 |
| 68.36261 | 16.48503 |
| 68.38294 | 17.89762 |
| 68.40326 | 18.57441 |
| 68.42358 | 17.54779 |
| 68.4439 | 18.47198 |
| 68.46422 | 19.43846 |
| 68.48454 | 18.36769 |
| 68.50486 | 15.75959 |
| 68.52518 | 14.93972 |
| 68.54551 | 18.00849 |
| 68.56583 | 17.56756 |
| 68.58615 | 11.85472 |
| 68.60647 | 9.60518 |
| 68.62679 | 9.4273 |
| 68.64711 | 10.99787 |
| 68.66743 | 8.71406 |
| 68.68775 | 10.54754 |
| 68.70808 | 9.0449 |
| 68.7284 | 7.78473 |
| 68.74872 | 6.17904 |
| 68.76904 | 4.66771 |
| 68.78936 | 5.43971 |
| 68.80968 | 1.80928 |
| 68.83 | 5.0605 |
| 68.85032 | 5.93042 |
| 68.87065 | 5.55613 |
| 68.89097 | 6.09351 |
| 68.91129 | 4.95657 |
| 68.93161 | 4.55851 |
| 68.95193 | 6.74899 |
| 68.97225 | 5.19718 |
| 68.99257 | 2.73511 |
| 69.01289 | 0.19723 |
| 69.03321 | 2.98051 |
| 69.05354 | 2.39666 |
| 69.07386 | 3.55565 |
| 69.09418 | 4.5646 |
| 69.1145 | 3.06626 |
| 69.13482 | 6.96549 |
| 69.15514 | 6.69084 |
| 69.17546 | 11.38431 |
| 69.19578 | 12.42898 |
| 69.21611 | 13.4281 |
| 69.23643 | 14.95643 |
| 69.25675 | 16.76138 |
| 69.27707 | 19.01981 |
| 69.29739 | 20.20255 |
| 69.31771 | 24.43248 |
| 69.33803 | 27.24991 |
| 69.35835 | 30.7702 |
| 69.37868 | 32.5294 |
| 69.399 | 31.88153 |
| 69.41932 | 29.95865 |
| 69.43964 | 31.40437 |
| 69.45996 | 30.79319 |
| 69.48028 | 31.12631 |
| 69.5006 | 27.79525 |
| 69.52092 | 28.00377 |
| 69.54125 | 28.17655 |
| 69.56157 | 28.31357 |
| 69.58189 | 28.41484 |
| 69.60221 | 28.48036 |
| 69.62253 | 28.51013 |
| 69.64285 | 28.50416 |
| 69.66317 | 28.46243 |
| 69.68349 | 28.38496 |
| 69.70381 | 28.27173 |
| 69.72414 | 28.12276 |
| 69.74446 | 27.93803 |
| 69.76478 | 27.71756 |
| 69.7851 | 27.46133 |
| 69.80542 | 27.16936 |
| 69.82574 | 26.84164 |
| 69.84606 | 26.47816 |
| 69.86638 | 26.07894 |
| 69.88671 | 25.64397 |
| 69.90703 | 25.17325 |
| 69.92735 | 24.66678 |
| 69.94767 | 24.12456 |
| 69.96799 | 23.54659 |
| 69.98831 | 22.93287 |
| 70.00863 | 22.2834 |

Fig 3. Spider plot of all the(a) treatments and (b) varieties on the morphological attributes of sesame plants.

|  | **Variety** | **Plant height** | **No. of leaves** | **Leaf area** | **No. of capsules** | **Stem diameter** | **Capsule weight** |
| --- | --- | --- | --- | --- | --- | --- | --- |
|  | **Max** | **90.000** | **50.000** | **15.000** | **25.000** | **4.000** | **0.850** |
|  | **Min** | **50.000** | **30.000** | **10.000** | **5.000** | **1.000** | **0.600** |
|  | Millennium | 82.733 | 33.667 | 12.733 | 21.733 | 2.299 | 0.811 |
| 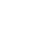  \|  \| \| --- \| | NS-2016 | 62.933 | 45.333 | 11.067 | 14.333 | 1.618 | 0.735 |
|  | Pearl | 55.333 | 46.200 | 11.400 | 7.067 | 2.438 | 0.736 |
|  | TH-6 | 84.467 | 39.933 | 14.733 | 17.600 | 2.386 | 0.762 |
|  | TS-5 | 68.400 | 46.200 | 14.333 | 15.200 | 3.158 | 0.753 |
|  |  |  |  |  |  |  |  |
|  | **Treatment** | **Plant height** | **No. of leaves** | **Leaf area** | **No. of capsules** | **Stem diameter** | **Capsule weight** |
|  | **Max** | **73.400** | **43.200** | **14.000** | **16.533** | **2.409** | **0.781** |
|  | **Min** | **69.067** | **41.400** | **10.600** | **14.400** | **2.357** | **0.733** |
|  | Control | 69.667 | 43.200 | 13.333 | 14.600 | 2.379 | 0.733 |
|  | ChNPs | 72.000 | 41.867 | 13.267 | 15.667 | 2.375 | 0.781 |
|  | ChNPs | 73.400 | 42.467 | 14.000 | 16.533 | 2.409 | 0.779 |
|  | Confidor | 69.067 | 41.400 | 13.067 | 14.733 | 2.357 | 0.749 |
|  | Confidor+Talstar | 69.733 | 42.400 | 10.600 | 14.400 | 2.379 | 0.756 |
|  |  |  |  |  |  |  |  |
| **Variety** | **Treatment** | **Plant height** | **No. of leaves** | **Leaf area** | **No. of capsules** | **Stem diameter** | **Capsule weight** |
| Millennium | Control | 79.333 | 35.000 | 12.000 | 22.000 | 2.303 | 0.823 |
|  | ChNPs | 82.000 | 33.333 | 13.000 | 23.333 | 2.270 | 0.813 |
|  | ChNPs | 90.000 | 34.667 | 13.667 | 22.333 | 2.327 | 0.900 |
|  | Confidor | 81.333 | 32.333 | 12.000 | 21.333 | 2.297 | 0.760 |
|  | Confidor+Talstar | 81.000 | 33.000 | 13.000 | 19.667 | 2.300 | 0.760 |
| NS-2016 | Control | 63.333 | 47.000 | 11.667 | 12.667 | 1.477 | 0.737 |
|  | ChNPs | 67.667 | 43.333 | 11.333 | 14.000 | 1.807 | 0.773 |
|  | ChNPs | 63.667 | 45.000 | 10.333 | 17.000 | 1.643 | 0.700 |
|  | Confidor | 59.667 | 47.000 | 11.333 | 12.667 | 1.533 | 0.737 |
|  | Confidor+Talstar | 60.333 | 44.333 | 10.667 | 15.333 | 1.630 | 0.730 |
| Pearl | Control | 56.000 | 48.667 | 11.333 | 7.000 | 2.507 | 0.660 |
|  | ChNPs | 53.333 | 46.667 | 11.667 | 7.333 | 2.407 | 0.800 |
|  | ChNPs | 58.000 | 44.333 | 11.000 | 7.000 | 2.447 | 0.710 |
|  | Confidor | 50.000 | 44.333 | 12.000 | 7.667 | 2.417 | 0.717 |
|  | Confidor+Talstar | 59.333 | 47.000 | 11.000 | 6.333 | 2.413 | 0.793 |
| TH-6 | Control | 81.333 | 39.667 | 16.667 | 17.000 | 2.383 | 0.720 |
|  | ChNPs | 85.667 | 39.000 | 14.667 | 17.667 | 2.393 | 0.743 |
|  | ChNPs | 88.000 | 42.333 | 18.000 | 20.333 | 2.357 | 0.800 |
|  | Confidor | 87.000 | 37.333 | 18.333 | 17.000 | 2.427 | 0.813 |
|  | Confidor+Talstar | 80.333 | 41.333 | 6.000 | 16.000 | 2.370 | 0.733 |
| TS-5 | Control | 68.333 | 45.667 | 15.000 | 14.333 | 3.227 | 0.723 |
|  | ChNPs | 71.333 | 47.000 | 15.667 | 16.000 | 2.997 | 0.773 |
|  | ChNPs | 67.333 | 46.000 | 17.000 | 16.000 | 3.270 | 0.787 |
|  | Confidor | 67.333 | 46.000 | 11.667 | 15.000 | 3.113 | 0.720 |
|  | Confidor+Talstar | 67.667 | 46.333 | 12.333 | 14.667 | 3.183 | 0.763 |
|  |  |  |  |  |  |  |  |
| **Treatment** | **Variety** | **Plant height** | **No. of leaves** | **Leaf area** | **No. of capsules** | **Stem diameter** | **Capsule weight** |
| Control | Millennium | 79.333 | 35.000 | 12.000 | 22.000 | 2.303 | 0.823 |
|  | NS-2016 | 63.333 | 47.000 | 11.667 | 12.667 | 1.477 | 0.737 |
|  | Pearl | 56.000 | 48.667 | 11.333 | 7.000 | 2.507 | 0.660 |
|  | TH-6 | 81.333 | 39.667 | 16.667 | 17.000 | 2.383 | 0.720 |
|  | TS-5 | 68.333 | 45.667 | 15.000 | 14.333 | 3.227 | 0.723 |
| ChNPs | Millennium | 82.000 | 33.333 | 13.000 | 23.333 | 2.270 | 0.813 |
|  | NS-2016 | 67.667 | 43.333 | 11.333 | 14.000 | 1.807 | 0.773 |
|  | Pearl | 53.333 | 46.667 | 11.667 | 7.333 | 2.407 | 0.800 |
|  | TH-6 | 85.667 | 39.000 | 14.667 | 17.667 | 2.393 | 0.743 |
|  | TS-5 | 71.333 | 47.000 | 15.667 | 16.000 | 2.997 | 0.773 |
| ChNPs | Millennium | 90.000 | 34.667 | 13.667 | 22.333 | 2.327 | 0.900 |
|  | NS-2016 | 63.667 | 45.000 | 10.333 | 17.000 | 1.643 | 0.700 |
|  | Pearl | 58.000 | 44.333 | 11.000 | 7.000 | 2.447 | 0.710 |
|  | TH-6 | 88.000 | 42.333 | 18.000 | 20.333 | 2.357 | 0.800 |
|  | TS-5 | 67.333 | 46.000 | 17.000 | 16.000 | 3.270 | 0.787 |
| Confidor | Millennium | 81.333 | 32.333 | 12.000 | 21.333 | 2.297 | 0.760 |
|  | NS-2016 | 59.667 | 47.000 | 11.333 | 12.667 | 1.533 | 0.737 |
|  | Pearl | 50.000 | 44.333 | 12.000 | 7.667 | 2.417 | 0.717 |
|  | TH-6 | 87.000 | 37.333 | 18.333 | 17.000 | 2.427 | 0.813 |
|  | TS-5 | 67.333 | 46.000 | 11.667 | 15.000 | 3.113 | 0.720 |
| Confidor+Talstar | Millennium | 81.000 | 33.000 | 13.000 | 19.667 | 2.300 | 0.760 |
|  | NS-2016 | 60.333 | 44.333 | 10.667 | 15.333 | 1.630 | 0.730 |
|  | Pearl | 59.333 | 47.000 | 11.000 | 6.333 | 2.413 | 0.793 |
|  | TH-6 | 80.333 | 41.333 | 6.000 | 16.000 | 2.370 | 0.733 |
|  | TS-5 | 67.667 | 46.333 | 12.333 | 14.667 | 3.183 | 0.763 |

Fig 5. Spider plot of all the (a) treatments and (b) varieties on the biochemical attributes of sesame plants.

|  | **Variety** | **Catalase** | **Polyphenol oxidase** | **Peroxidase** | **Chlorophyll a** | **Chlorophyll b** | **Total Chlorophyll** | **Phenylalanine ammonia lyase** |
| --- | --- | --- | --- | --- | --- | --- | --- | --- |
|  | **Max** | **3.500** | **140.000** | **20.000** | **80.000** | **110.000** | **20.000** | **4.000** |
|  | **Min** | **1.500** | **100.000** | **1.000** | **35.000** | **40.000** | **12.000** | **0.500** |
|  | Millennium | 2.338 | 105.237 | 13.124 | 74.733 | 102.359 | 19.177 | 2.363 |
| 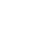  \|  \| \| --- \| | NS-2016 | 1.779 | 112.691 | 2.427 | 70.821 | 95.627 | 13.180 | 0.930 |
|  | Pearl | 2.178 | 132.399 | 2.410 | 39.831 | 46.529 | 14.811 | 3.072 |
|  | TH-6 | 3.033 | 117.550 | 18.472 | 65.213 | 84.223 | 18.522 | 1.376 |
|  | TS-5 | 2.045 | 136.677 | 2.155 | 58.209 | 75.687 | 16.209 | 3.691 |
|  |  |  |  |  |  |  |  |  |
|  | **Treatment** | **Catalase** | **Polyphenol oxidase** | **Chlorophylla** | **Chlorophyll a** | **Chlorophyll b** | **Total Chlorophyll** | **Phenylalanine ammonia lyase** |
|  | **Max** | **3.173** | **135.093** | **18.972** | **64.173** | **84.940** | **18.053** | **3.507** |
|  | **Min** | **1.483** | **104.031** | **1.611** | **56.595** | **70.658** | **15.487** | **1.127** |
|  | Control | 2.067 | 127.133 | 13.865 | 60.249 | 83.842 | 16.130 | 3.507 |
|  | ChNPs | 1.483 | 130.435 | 1.611 | 64.173 | 84.940 | 16.133 | 2.697 |
|  | ChNPs | 3.173 | 104.031 | 1.980 | 63.991 | 82.732 | 18.053 | 1.433 |
|  | Confidor | 2.094 | 107.862 | 2.158 | 63.799 | 82.252 | 16.095 | 1.127 |
|  | Confidor+Talstar | 2.556 | 135.093 | 18.972 | 56.595 | 70.658 | 15.487 | 2.668 |
|  |  |  |  |  |  |  |  |  |
| **Variety** | **Treatment** | **Catalase** | **Polyphenol oxidase** | **Chlorophylla** | **Chlorophyll a** | **Chlorophyll b** | **Total Chlorophyll** | **Phenylalanine ammonia lyase** |
| Millennium | Control | 2.137 | 108.750 | 58.837 | 60.607 | 95.307 | 17.087 | 5.150 |
|  | ChNPs | 1.573 | 117.820 | 0.950 | 89.500 | 125.617 | 15.707 | 3.067 |
|  | ChNPs | 4.700 | 63.217 | 2.123 | 74.333 | 87.173 | 24.400 | 2.037 |
|  | Confidor | 2.450 | 114.623 | 2.334 | 74.630 | 104.303 | 22.023 | 1.130 |
|  | Confidor+Talstar | 0.830 | 121.773 | 1.374 | 74.593 | 99.393 | 16.667 | 0.430 |
| NS-2016 | Control | 1.800 | 102.017 | 1.934 | 81.273 | 136.100 | 16.690 | 0.853 |
|  | ChNPs | 0.850 | 142.780 | 1.827 | 70.223 | 90.480 | 12.327 | 0.473 |
|  | ChNPs | 1.273 | 87.510 | 3.973 | 66.940 | 91.767 | 12.870 | 0.807 |
|  | Confidor | 2.200 | 99.297 | 2.880 | 75.857 | 86.597 | 12.943 | 0.170 |
|  | Confidor+Talstar | 2.773 | 131.850 | 1.519 | 59.813 | 73.193 | 11.070 | 2.347 |
| Pearl | Control | 2.367 | 147.333 | 5.983 | 54.460 | 66.520 | 14.187 | 4.960 |
|  | ChNPs | 1.010 | 134.510 | 1.912 | 45.177 | 51.040 | 16.617 | 1.697 |
|  | ChNPs | 5.133 | 109.537 | 1.578 | 35.983 | 44.727 | 14.560 | 0.930 |
|  | Confidor | 1.590 | 130.247 | 1.585 | 33.553 | 35.970 | 11.153 | 1.100 |
|  | Confidor+Talstar | 0.790 | 140.367 | 0.993 | 29.980 | 34.387 | 17.537 | 6.673 |
| TH-6 | Control | 2.203 | 133.843 | 0.394 | 67.477 | 76.450 | 18.037 | 1.063 |
|  | ChNPs | 2.763 | 127.940 | 0.533 | 51.660 | 71.703 | 18.127 | 0.603 |
|  | ChNPs | 3.123 | 119.077 | 1.447 | 66.477 | 87.673 | 19.157 | 1.613 |
|  | Confidor | 3.193 | 57.020 | 1.405 | 79.263 | 104.630 | 17.330 | 0.950 |
|  | Confidor+Talstar | 3.880 | 149.870 | 88.580 | 61.190 | 80.657 | 19.960 | 2.650 |
| TS-5 | Control | 1.827 | 143.723 | 2.178 | 37.427 | 44.833 | 14.650 | 5.510 |
|  | ChNPs | 1.220 | 129.123 | 2.835 | 64.303 | 85.860 | 17.890 | 7.643 |
|  | ChNPs | 1.637 | 140.813 | 0.781 | 76.223 | 102.320 | 19.280 | 1.780 |
|  | Confidor | 1.037 | 138.123 | 2.586 | 55.693 | 79.760 | 17.027 | 2.283 |
|  | Confidor+Talstar | 4.507 | 131.603 | 2.395 | 57.400 | 65.660 | 12.200 | 1.240 |
|  |  |  |  |  |  |  |  |  |
| **Treatment** | **Variety** | **Catalase** | **Polyphenol oxidase** | **Chlorophylla** | **Chlorophyll a** | **Chlorophyll b** | **Total Chlorophyll** | **Phenylalanine ammonia lyase** |
| Control | Millennium | 2.137 | 108.750 | 58.837 | 60.607 | 95.307 | 17.087 | 5.150 |
|  | NS-2016 | 1.800 | 102.017 | 1.934 | 81.273 | 136.100 | 16.690 | 0.853 |
|  | Pearl | 2.367 | 147.333 | 5.983 | 54.460 | 66.520 | 14.187 | 4.960 |
|  | TH-6 | 2.203 | 133.843 | 0.394 | 67.477 | 76.450 | 18.037 | 1.063 |
|  | TS-5 | 1.827 | 143.723 | 2.178 | 37.427 | 44.833 | 14.650 | 5.510 |
| ChNPs | Millennium | 1.573 | 117.820 | 0.950 | 89.500 | 125.617 | 15.707 | 3.067 |
|  | NS-2016 | 0.850 | 142.780 | 1.827 | 70.223 | 90.480 | 12.327 | 0.473 |
|  | Pearl | 1.010 | 134.510 | 1.912 | 45.177 | 51.040 | 16.617 | 1.697 |
|  | TH-6 | 2.763 | 127.940 | 0.533 | 51.660 | 71.703 | 18.127 | 0.603 |
|  | TS-5 | 1.220 | 129.123 | 2.835 | 64.303 | 85.860 | 17.890 | 7.643 |
| ChNPs | Millennium | 4.700 | 63.217 | 2.123 | 74.333 | 87.173 | 24.400 | 2.037 |
|  | NS-2016 | 1.273 | 87.510 | 3.973 | 66.940 | 91.767 | 12.870 | 0.807 |
|  | Pearl | 5.133 | 109.537 | 1.578 | 35.983 | 44.727 | 14.560 | 0.930 |
|  | TH-6 | 3.123 | 119.077 | 1.447 | 66.477 | 87.673 | 19.157 | 1.613 |
|  | TS-5 | 1.637 | 140.813 | 0.781 | 76.223 | 102.320 | 19.280 | 1.780 |
| Confidor | Millennium | 2.450 | 114.623 | 2.334 | 74.630 | 104.303 | 22.023 | 1.130 |
|  | NS-2016 | 2.200 | 99.297 | 2.880 | 75.857 | 86.597 | 12.943 | 0.170 |
|  | Pearl | 1.590 | 130.247 | 1.585 | 33.553 | 35.970 | 11.153 | 1.100 |
|  | TH-6 | 3.193 | 57.020 | 1.405 | 79.263 | 104.630 | 17.330 | 0.950 |
|  | TS-5 | 1.037 | 138.123 | 2.586 | 55.693 | 79.760 | 17.027 | 2.283 |
| Confidor+Talstar | Millennium | 0.830 | 121.773 | 1.374 | 74.593 | 99.393 | 16.667 | 0.430 |
|  | NS-2016 | 2.773 | 131.850 | 1.519 | 59.813 | 73.193 | 11.070 | 2.347 |
|  | Pearl | 0.790 | 140.367 | 0.993 | 29.980 | 34.387 | 17.537 | 6.673 |
|  | TH-6 | 3.880 | 149.870 | 88.580 | 61.190 | 80.657 | 19.960 | 2.650 |
|  | TS-5 | 4.507 | 131.603 | 2.395 | 57.400 | 65.660 | 12.200 | 1.240 |
